# Effects of mesophyll conductance on vegetation responses to elevated CO_2_ concentrations in a land surface model

**DOI:** 10.1111/gcb.14604

**Published:** 2019-03-23

**Authors:** Jürgen Knauer, Sönke Zaehle, Martin G. De Kauwe, Nur H. A. Bahar, John R. Evans, Belinda E. Medlyn, Markus Reichstein, Christiane Werner

**Affiliations:** ^1^ Department of Biogeochemical Integration Max Planck Institute for Biogeochemistry Jena Germany; ^2^ International Max Planck Research School for Global Biogeochemical Cycles (IMPRS gBGC) Jena Germany; ^3^ Michael‐Stifel Center Jena for Data‐Driven and Simulation Science Jena Germany; ^4^ ARC Centre of Excellence for Climate Extremes and the Climate Change Research Centre University of New South Wales Sydney NSW Australia; ^5^ ARC Centre of Excellence in Plant Energy Biology, Division of Plant Sciences Research School of Biology, Australian National University Canberra ACT Australia; ^6^ ARC Centre of Excellence for Translational Photosynthesis, Division of Plant Sciences Research School of Biology, Australian National University Canberra ACT Australia; ^7^ Hawkesbury Institute for the Environment Western Sydney University Richmond NSW Australia; ^8^ Department of Ecosystem Physiology University of Freiburg Freiburg Germany

**Keywords:** elevated CO_2_ concentrations, land surface modeling, mesophyll conductance, photosynthetic CO_2_ sensitivity, representative concentration pathways

## Abstract

Mesophyll conductance (*g*_m_) is known to affect plant photosynthesis. However, *g*_m_ is rarely explicitly considered in land surface models (LSMs), with the consequence that its role in ecosystem and large‐scale carbon and water fluxes is poorly understood. In particular, the different magnitudes of *g*_m_ across plant functional types (PFTs) are expected to cause spatially divergent vegetation responses to elevated CO_2_ concentrations. Here, an extensive literature compilation of *g*_m_ across major vegetation types is used to parameterize an empirical model of *g*_m_ in the LSM JSBACH and to adjust photosynthetic parameters based on simulated *A*_n_ − *C*_i_ curves. We demonstrate that an explicit representation of *g*_m_ changes the response of photosynthesis to environmental factors, which cannot be entirely compensated by adjusting photosynthetic parameters. These altered responses lead to changes in the photosynthetic sensitivity to atmospheric CO_2_ concentrations which depend both on the magnitude of *g*_m_ and the climatic conditions, particularly temperature. We then conducted simulations under ambient and elevated (ambient + 200 μmol/mol) CO_2_ concentrations for contrasting ecosystems and for historical and anticipated future climate conditions (representative concentration pathways; RCPs) globally. The *g*_m_‐explicit simulations using the RCP8.5 scenario resulted in significantly higher increases in gross primary productivity (GPP) in high latitudes (+10% to + 25%), intermediate increases in temperate regions (+5% to + 15%), and slightly lower to moderately higher responses in tropical regions (−2% to +5%), which summed up to moderate GPP increases globally. Similar patterns were found for transpiration, but with a lower magnitude. Our results suggest that the effect of an explicit representation of *g*_m_ is most important for simulated carbon and water fluxes in the boreal zone, where a cold climate coincides with evergreen vegetation.

## 1 INTRODUCTION

The representation of photosynthesis in land surface models (LSMs) is critical for simulating the response of the terrestrial biosphere to global environmental change (Booth et al., 2012; Rogers et al., 2017), the land uptake of CO_2_, as well as the coupling of the water and carbon cycles. The photosynthesis schemes embedded within state‐of‐the‐art LSMs commonly assume that the CO_2_ concentration available for carboxylation equals the CO_2_ concentration in the sub‐stomatal cavity, that is the intercellular CO_2_ concentration (*C*_i_). This corresponds to the assumption that the conductance to CO_2_ transfer within the leaf (mesophyll conductance, *g*_m_) is infinite, and that the CO_2_ concentration at the actual place of carboxylation in the chloroplast stroma (chloroplastic CO_2_ concentration, *C*_c_) equals *C*_i_. However, evidence has clearly shown that *g*_m_ is finite (Flexas, Ribas‐Carbó, Diaz‐Espejo, Galmés, & Medrano, 2008; Warren, 2008) and that it causes a clear drawdown of the CO_2_ concentration between the sub‐stomatal cavity and the chloroplast stroma. The magnitude of this drawdown depends both on *g*_m_ and the photosynthetic capacity of the leaf, which is reflected in the definition of *g*_m_: *g*_m_ = *A*_n_/(*C*_i_ − *C*_c_), where *A*_n_ is net assimilation. Replacing *C*_i_ with *C*_c_ as the available CO_2_ concentration for photosynthesis has been shown to change the response of simulated photosynthesis to environmental drivers (Niinemets, Díaz‐Espejo, Flexas, Galmés, & Warren, 2009), which has important implications for large‐scale simulations of land carbon uptake (Sun, Gu, Dickinson, Norby et al., 2014).

*g*_m_ is a complex physiological property which integrates several leaf‐internal sub‐conductances in both the gaseous and liquid phase, including the intercellular airspace, cell walls, plasma membranes, cytoplasm, and the chloroplast envelopes and stroma (Evans, Kaldenhoff, Genty, & Terashima, 2009). *g*_m_ is known to change dynamically in response to several environmental stimuli at the time scale of minutes (Warren, 2008). At the same time, its absolute magnitude is constrained by leaf anatomical and structural traits (e.g. cell wall thickness, chloroplast surface area attached to the intercellular airspaces (Tomás et al., 2013)), with the consequence that the values of *g*_m_ differ considerably among vegetation types (Flexas et al., 2008).

Despite its important role in photosynthesis and the distinct differences across plant functional types (PFTs), *g*_m_ is at present not explicitly considered in the vast majority of LSMs for two main reasons: (1) the current process understanding of *g*_m_ is severely limited (Rogers et al., 2017) as its response to environmental drivers, foremost light and CO_2_ concentration but also temperature, is largely unknown and currently an area of intensive research (von Caemmerer & Evans, 2015; Gu & Sun, 2014; Tazoe, Caemmerer, Badger, & Evans, 2009; Théroux‐Rancourt & Gilbert, 2017; Xiong et al., 2015), and (2) the effects of *g*_m_ are implicitly included in current models since the overestimation of CO_2_ available for photosynthesis is compensated for by an underestimated (apparent) photosynthetic capacity. This means that parameters representing photosynthetic capacity, which are currently estimated on a *C*_i_‐basis, would need to be re‐estimated on a *C*_c_‐basis if *g*_m_ were to be explicitly considered in models (Sun, Gu, Dickinson, Pallardy et al., 2014).

It is likely for these two complications that so far only one study (Sun, Gu, Dickinson, Norby et al., 2014) focused on the effects of an explicit representation of *g*_m_ in a LSM (the Community Land Model 4.5). Sun, Gu, Dickinson, Norby et al. (2014) showed that the overestimation of the available CO_2_ concentration for photosynthesis due to the assumption of an infinite *g*_m_ leads to an underestimation of the photosynthetic sensitivity to rising atmospheric CO_2_ concentrations (*C*_a_). As a consequence, replacing the implicit simulation of *g*_m_ with an explicit one significantly increased the responsiveness of GPP (+16% from 1901 to 2010) to rising *C*_a_ as long as *C*_a_ was not saturating.

The stronger response of photosynthesis to rising atmospheric CO_2_ concentrations with an explicitly modeled *g*_m_ as shown in the study by Sun, Gu, Dickinson, Norby et al. (2014) implies that the physiological responses to rising atmospheric CO_2_ concentrations will vary among plant groups that have intrinsically different values of *g*_m_. Consequently, it might be hypothesized that photosynthesis of plants with a low *g*_m_ (e.g. evergreen species) are more responsive to rising atmospheric CO_2_ concentrations than plants with a higher *g*_m_ (e.g. herbaceous plants), which potentially gives the former plant group a relative advantage over the latter in a high CO_2_ world (Niinemets, Flexas, & Peñuelas, 2011). A stronger response of photosynthesis to *C*_a_ is likely to also affect stomatal conductance (*g*_s_) given that *g*_s_ and *A*_n_ are tightly coupled (Wong, Cowan, & Farquhar, 1979). As a consequence, the consideration of *g*_m_ is expected to have important implications for both terrestrial carbon and water fluxes, as well as their coupling (e.g. water‐use efficiency, Flexas et al., 2016). Such plant type‐specific physiological responses would thus not only have important implications for the future global distribution of vegetation types, but also for large‐scale patterns of biogeochemical cycles and associated physical climate feedbacks (e.g. evaporative cooling).

In this paper, we explore whether *g*_m_ has implications for simulations of future global carbon and water fluxes, and to what extent the effects are expected to differ among vegetation types and climatic conditions. In the following, we (1) compile a global database of *g*_m_ measurements, (2) describe the *g*_m_ model and its incorporation into the LSM JSBACH (Knauer, Werner, & Zaehle, 2015; Reick, Raddatz, Brovkin, & Gayler, 2013), (3) outline the model parameterization and the necessary adjustment of photosynthetic parameters, (4) analyze the effects of an explicit *g*_m_ on the photosynthetic sensitivity to *C*_a_ at the leaf‐ and ecosystem level, and (5) investigate its relevance for future carbon and water fluxes globally.

## 2 METHODS

To investigate the effects of *g*_m_ on simulations of water and carbon fluxes, we tested two different approaches in the LSM JSBACH:

**Implicit *g*_m_:** Effects of *g*_m_ are considered implicitly by employing apparent (*C*_i_‐based) photosynthetic parameters. This represents the current scenario in most LSMs. Rubisco kinetic parameters were taken from Bernacchi et al. (2001). This model version is denoted as ***Imp***.

**Explicit *g*_m_**:* g*_m_ is modeled explicitly as described in Section 2.1. Rubisco kinetic parameters were taken from Bernacchi et al. (2002), and were determined on a *C*_c_‐basis. Four sub‐versions (denoted as ***Exp***,***ExpC***,***ExpL***,***ExpCL***) were implemented, which differ with respect to whether *g*_m_ is affected by *C*_i_ and/or light (Table 1). The effect of these two factors is contentious in the literature (Gu & Sun, 2014; Théroux‐Rancourt & Gilbert, 2017), hence it is relevant to investigate their potential sensitivities to simulations of photosynthesis at the leaf to the global scale.

**Table 1:** Environmental responses considered in the *g*_m_ model versions implemented in this study

Model version	Temperature	Soil moisture	Canopy profile	Intercellular CO_2_ concentration	Light
*Exp*	x	x	x		
*ExpC*	x	x	x	x	
*ExpL*	x	x	x		x
*ExpCL*	x	x	x	x	x

Note that the two approaches differ only in the consideration of *g*_m_ (included implicitly or explicitly) and the Rubisco kinetic parameters (Michaelis‐Menten constants for CO_2_ (*K*_c_) and O_2_ (*K*_o_), photorespiratory CO_2_ compensation point (Γ*)) as well as their temperature responses (see Appendix S1 for model formulations and Table S1 for parameter values).

### 2.1 Mesophyll conductance model

The *g*_m_ model implemented here is a multiplicative formulation, in which a PFT‐specific maximum (i.e. unstressed) value of *g*_m_ at the reference temperature of 25°C (*g*_m,max25_) is modified by environmental factors:

(1) $$g_{m}=\text{max}\left(g_{m,\min},g_{m,\max25}f_{1}\left(N\right)f_{2}\left(T_{l}\right)f_{3}\left(\theta \right)f_{4}\left(C_{i}\right)f_{5}\left(Q_{a}\right)\right)$$

where N is leaf nitrogen content, *T_l_* is leaf temperature, θ is soil moisture content, *C*_i_ is intercellular CO_2_ concentration, *Q*_a_ is absorbed photosynthetic photon flux density, and *f* denotes “function of”. *g*_m,min_ is defined as g_m,min_ = *f*_min_ × *g*_m,max25_, and accounts for the fact that *g*_m_ does not decrease to zero even under unfavorable conditions such as severe water stress (e.g. Galmés, Medrano, & Flexas, 2007). *f*_min_ was parameterized from data presented in Delfine, Loreto, Pinelli, Tognetti, and Alvino (2005), Galmés, Abadía, Medrano, and Flexas (2007), and Galmés, Medrano et al. (2007) as *f*_min_ = 0.15. In Equation (1), *g*_m,max25_ and *f*_1_ represent leaf structural determinants of *g*_m_, whereas *f*_2_ − *f*_5_ describe instantaneous physiological responses. Note that the last two terms in Equation (1) (*f*_4_ and *f*_5_) are only considered in some model versions (Table 1). Acclimation of *g*_m_ to elevated CO_2_ was not considered in the model as measured *g*_m_ of plants grown under ambient and elevated CO_2_ concentrations did not show consistent differences (Kitao et al., 2015; Mizokami, Noguchi, Kojima, Sakakibara, & Terashima, 2018; Singsaas, Ort, & Delucia, 2003).

#### 2.1.1 Canopy profile

*g*_m_ generally declines with depth through the canopy, and is usually higher in sun than in shade leaves (Hanba, Kogami, & Terashima, 2002; Piel, Frak, Roux, & Genty, 2002). It has been found that *g*_m_ varies in a similar manner to photosynthetic capacity (or N) across the canopy profile (Montpied, Granier, & Dreyer, 2009). This decline with canopy depth might be related to the relatively low mesophyll thickness and the lower chloroplast surface area exposed to the intercellular airspaces in shade‐adapted leaves (Evans, Caemmerer, Setchell, & Hudson, 1994; Hanba et al., 2002). Here, we implemented the following canopy profile of *g*_m_:

(2) $$f_{1}\left(N\right)=e^{-k_{n}L}$$

where *k*_n_ is the canopy nitrogen extinction coefficient and *L* is the leaf area index (LAI). *k*_n_ was assumed to be 0.11 following Zaehle and Friend (2010). Thus, the canopy gradient of *g*_m_ equals the one of *V*_cmax_ and *J*_max_ in the model. Such a behavior was confirmed by several studies (Han, Iio, Naramoto, & Kakubari, 2010; Montpied et al., 2009; Warren, Löw, Matyssek, & Tausz, 2007), but also higher (Zhang & Yin, 2012) and lower (Cano et al., 2011; Niinemets, Cescatti, Rodeghiero, & Tosens, 2006) gradients for *g*_m_ compared to *V*_cmax_ have been found, suggesting that *k*_n_ is site‐ and probably PFT‐specific (Warren et al., 2007).

#### 2.1.2 Temperature response

The temperature response of *g*_m_ is the result of different physical and physiological processes in mesophyll cells (e.g. solubility and diffusivity of CO_2_ in water), and the response is likely to differ across cell compartments, for example, membranes and cell walls (Evans & von Caemmerer, 2013). The overall response of *g*_m_ to leaf temperature can be described by a modified Arrhenius function (Johnson, Eyring, & Williams, 1942):

(3) $$f_{2}\left(T_{l}\right)=exp\left(\frac{H_{a}\left(T_{l}-T_{\text{ref}}\right)}{T_{\text{ref}}RT_{l}}\right)\frac{1+exp\left(\frac{T_{\text{ref}}\Delta S-H_{d}}{T_{\text{ref}}R}\right)}{1+exp\left(\frac{T_{l}\Delta S-H_{d}}{T_{l}R}\right)}$$

where *H*_a_ is the activation energy (J/mol), *H*_d_ is the deactivation energy (J/mol), ΔS is the entropy term (J mol^−1^ K^−1^) (see Table S1 for parameter values), *T*_l_ is the leaf temperature (K), *T*_ref_ is the reference temperature (298.15 K), and *R* is the universal gas constant (8.314 J mol^−1^ K^−1^). Equation (3) was parameterized according to Bernacchi, Portis, Nakano, Caemmerer, and Long (2002) and shows a temperature optimum close to 35.5°C. The use of the parameter values reported in Bernacchi et al. (2002) is consistent with the *C*_c_‐based Rubisco kinetic parameters used in this study (*K*_o,Cc_, *K*_c,Cc_, $\Gamma_{\mathrm{Cc}}^{*}$, Appendix S1), which were derived assuming the same temperature response of *g*_m_ (Equation (3)). Published temperature responses of *g*_m_ differ with respect to the behavior at high temperatures, and both hump‐shaped (Egea, González‐Real, Baille, Nortes, & Diaz‐Espejo, 2011), as well as monotonously increasing responses (Scafaro, Caemmerer, Evans, & Atwell, 2011) have been documented. Similarly, *H*_a_ is likely to be species‐specific (Walker, Ariza, Kaines, Badger, & Cousins, 2013), though no clear patterns across species and growth conditions have been identified (von Caemmerer & Evans, 2015). Thus, parameters in Equation (3) were assumed to be identical for all vegetation types.

#### 2.1.3 Soil moisture response

The decline of *g*_m_ with increasing soil water stress has been widely reported (e.g. Galmés, Medrano et al., 2007; Misson, Limousin, Rodriguez, & Letts, 2010; Varone et al., 2012), and has been attributed to the role of aquaporins in leaf‐internal CO_2_ transport (Miyazawa, Yoshimura, Shinzaki, Maeshima, & Miyake, 2008; Perez‐Martin et al., 2014). Here, we implemented the following soil moisture dependence of *g*_m_:

(4) $$f_{3}\left(\theta \right)=\left\{\begin{array}{c} 1\\ {\left[\frac{\theta -\theta_{\mathrm{wilt}}}{\theta_{\text{crit}}-\theta_{\mathrm{wilt}}}\right]}^{q_{m}}\\ 0 \end{array}\right.\begin{array}{c} \theta \geq \theta_{\mathrm{crit}}\\ \theta <\theta_{\mathrm{crit}}\\ \theta \leq \theta_{\mathrm{wilt}} \end{array}$$

where θ is soil moisture (m), $\theta_{wilt}$ is the permanent wilting point (m), below which water stress is at its maximum, and $\theta_{crit}$ is the critical soil moisture content (m), which marks the onset of soil water stress. $\theta_{wilt}$ and $\theta_{crit}$ are constant fractions (0.32 and 0.7 for $\theta_{wilt}$ and $\theta_{crit}$, respectively) of the field capacity, which is calculated depending on the grain size distribution of the soil. Equation (4) is applied to *g*_m_, *g*_s_, and leaf biochemistry (*V*_cmax_ and *J*_max_) but with different sensitivities (i.e. different values of the exponent *q*, i.e. *q*_m_, *q*_s_, and *q*_b_ for *g*_m_, *g*_s_, and leaf biochemistry, respectively). Using the same formulation, Egea, Verhoef, and Vidale (2011) found that imposing the highest sensitivity to *g*_m_, then to *g*_s_, and finally to *V*_cmax_ and *J*_max_ best captured the behavior of photosynthesis under water stressed conditions for a variety of species from different PFTs. The *q* parameters were defined accordingly as *q*_m_ = 0.75, *q*_s_ = 0.50, and *q*_b_ = 0.25 for all PFTs.

#### 2.1.4 Response to intercellular CO_2_ concentration

Most studies investigating the response of *g*_m_ to *C*_i_ have found a continuous decrease of *g*_m_ with increasing *C*_i_ under field conditions (i.e. *C*_i_ above c. 200 μmol/mol) (Flexas et al., 2007; Hassiotou, Ludwig, Renton, Veneklaas, & Evans, 2009; Xiong et al., 2015), but see Tazoe et al., 2009). However, there is currently no physiological explanation to link the response of *g*_m_ to *C*_i_, and some concerns on the reliability of these measurements have been raised (Gu & Sun, 2014). We have implemented a *C*_i_ response function which was derived empirically based on leaf‐level measurements as shown in Figure S1.

(5) $$f_{4}\left(C_{i}\right)=f_{min}+1.5\left(1-e^{\left({-C}_{i}/38\right)}\right)e^{\left({-C}_{i}/460\right)}$$

Equation (5) describes an abrupt increase of *g*_m_ at low *C*_i_ (until approx. 100 μmol/mol), and an exponential decline thereafter (Figure S1).

#### 2.1.5 Light response

The effect of absorbed radiation on *g*_m_ and the underlying mechanisms driving this potential response are currently unresolved. Studies in which *g*_m_ was measured at different light levels have reported either no clear responses of *g*_m_ to variations in light (Loucos, Simonin, & Barbour, 2017; Tazoe et al., 2009; Yamori, Evans, & Caemmerer, 2010), or clear increases with light (Cai, Yang, Li, Wang, & Huang, 2017; Douthe, Dreyer, Brendel, & Warren, 2012; Yin et al., 2009) (see Figure S2). The following function was used to simulate the potential effects of light on g_m_:

(6) $$f_{5}\left(Q_{a}\right)=1-\left(1-f_{min}\right)e^{-0.003Q_{a}}$$

where *Q*_a_ is absorbed photosynthetic photon flux density (μmol m^−2^ s^−1^). Equation (6) corresponds to a light response curve that takes the values of approximately *f*_min_ and approximately 1 at *Q*_a_ values of 0 and 1,500 μmol m^−2^ s^−1^, respectively (see Figure S2). The function corresponds to a steep increase of *g*_m_ at low *Q*_a_, a value of 0.8 (relative to g_m,max25_) at approximately 500 μmol m^−2^ s^−1^ and a shallow response at high *Q*_a_.

### 2.2 C4 plants

**Figure 1:**
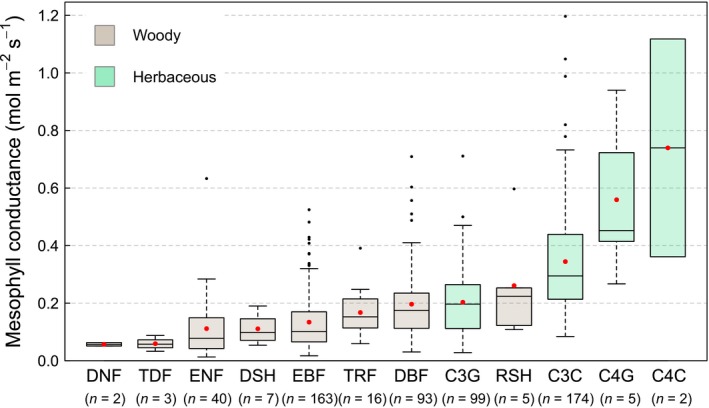
Maximum (unstressed) mesophyll conductance values for different plant functional types (PFTs), standardized to 25°C. Horizontal lines within boxes represent medians, red dots represent means, the lower and upper boundaries of the boxes represent the first and third quantile, respectively, and whiskers represent 1.5 times the interquartile range. PFT abbreviations are: DNF = deciduous needle‐leaf trees, TDF = tropical deciduous trees, ENF = evergreen needle‐leaf trees, DSH = deciduous shrubs, EBF = evergreen broadleaf trees/shrubs, TRF = tropical evergreen trees, DBF = deciduous broadleaf trees, C3G = C3 herbs and grasses, RSH = raingreen shrubs, C3C = C3 crops, C4G = C4 grasses and herbs, C4C = C4 crops. Data presented here were not standardized to a given *C*_i_ or to high light, and can be found in Appendix S3

In C4 plants, *g*_m_ describes the conductance to CO_2_ transfer from the intercellular airspace to the cytosol of the mesophyll cells, where the first binding of CO_2_ occurs. Thus, the main difference to C3 plants is that the chloroplast components are not part of the diffusion pathway (von Caemmerer & Furbank, 1999). This means that *g*_m_ in C4 plants causes a CO_2_ concentration drawdown from *C_i_* to *C*_m_, the CO_2_ concentration in the mesophyll cytosol (i.e. *g*_m_ = *A*_n_/(*C*_i_ − *C*_m_)). Recent methodological advances have enabled measurements of *g*_m_ in C4 plants (Barbour, Evans, Simonin, & Caemmerer, 2016; Ubierna, Gandin, Boyd, & Cousins, 2017). These measurements indicate that *g*_m_ is higher in C4 plants than in C3 plants (see Figure 1). The response of *g*_m_ to environmental factors was assumed to be identical to that in C3 plants (Equations (2), (3), (4), (5), (6)). This assumption could not be confirmed due to the scarcity of *g*_m_ measurements in C4 plants, but recent studies indicate that the temperature as well as the *C*_i_ response are qualitatively similar to that in C3 plants (Kolbe & Cousins, 2018; Ubierna et al., 2017). The relationship among C4 photosynthetic parameters was kept as in von Caemmerer and Furbank (1999) (see Table S1).

### 2.3 Implementation into the LSM JSBACH

The developed *g*_m_ model was incorporated into the LSM JSBACH (Knauer et al., 2015; Reick et al., 2013), which is the land component of the MPI Earth system model (Giorgetta et al., 2013). Vegetation in JSBACH is classified into PFTs, which may co‐occur in model grid cells as tiles, and which differ with respect to key physiological and biophysical properties. Fluxes and conductances are scaled to canopy‐level with the LAI for each PFT, and the cover fraction‐weighted mean of all tiles gives the respective grid cell value. Land‐atmosphere water fluxes are calculated with a bulk transfer approach (Schulz, Dümenil, & Polcher, 2001). Canopy radiative transfer is modeled as described in Wang (2003) based on the model of Goudriaan (1977) and considers sun‐lit and shaded canopy fractions in nine vertical layers. *g*_s_ is modeled according to Medlyn et al. (2011) with PFT‐specific stomatal slope parameters (*g*_1_) taken from Lin et al. (2015) and a constant residual stomatal conductance (*g*_0_) of 0.005 mol m^−2^ s^−1^. *A*_n_ is simulated according to Farquhar, Caemmerer, and Berry (1980) and von Caemmerer and Furbank (1999) for C3 and C4 vegetation, respectively, and photosynthetic capacity is taken from Kattge, Knorr, Raddatz, and Wirth (2009), and if applicable re‐calculated based on N_a_ (leaf nitrogen per area) data in Kattge et al. (2011). In the photosynthesis routine of the model, *g*_m_ is calculated first according to Equation (1), and *A*_n_, *g*_s_, *C*_i_, and *C*_c_ are subsequently solved iteratively. In the model versions where *g*_m_ depends on *C*_i_ (*ExpC* and *ExpCL*), *g*_m_ is first calculated according to Equation (1) with *f*_4_ set to 1 and then iteratively adjusted for *C*_i_ (Equation (5)) in the same loop where *A*_n_, *g*_s_, *C*_i_, and *C*_c_ are solved.

### 2.4 Maximum mesophyll conductance values (*g*_m,max25_)

To parameterize the key parameter in the model, *g*_m,max25_ (Equation (1)), we compiled an extensive literature review of leaf‐level *g*_m_‐measurements as described in Appendix S2. This dataset (Appendix S3) adds substantial new data to previous databases (e.g. Flexas et al., 2008) and comprises 609 individual *g*_m_ measurements of 319 species from 295 studies. Measurements were performed using all common methods used to estimate *g*_m_ (see e.g. Pons et al., 2009) and represent unstressed, fully expanded, and sun‐exposed leaves. If necessary, measurements were converted to units of mol m^−2^ s^−1^ and standardized to 25°C using Equation (3). If *g*_m_ was assumed to be light‐dependent (model versions *ExpL* and *ExpCL*), measurements were standardized to high light (1,500 μmol m^−2^ s^−1^) according to Equation (6). If *g*_m_ was assumed to be *C*_i_ dependent (model versions *ExpC* and *ExpCL*), *g*_m,max25_ was adjusted according to the *C*_i_ measured along with *g*_m_ (Equation (5)). This adjustment accounts for the fact that different vegetation types operate at different *C*_i_/*C*_a_ (depending on the stomatal behavior in the model (*g*_0_ and *g*_1_ parameters) and the vapor pressure deficit (VPD)). The *g*_m_ values were assigned to PFTs and the mean, median and standard error of the median were calculated (see Figure 1, Table 3).

### 2.5 Adjustment of *C*_i_‐based to *C*_c_‐based photosynthetic parameters

The explicit representation of *g*_m_ in photosynthesis models requires that photosynthetic parameters represent *C*_c_‐based rather than *C*_i_‐based values, as the latter implicitly include the effects of *g*_m_ (Ethier & Livingston, 2004). This typically requires that existing (i.e. *C*_i_‐based) parameters are adjusted to *C*_c_‐based parameters. Previous approaches for this parameter adjustment focused on the simultaneous derivation of *g*_m_, *V*_cmax_ and *J*_max_ from *A*_n_ − *C*_i_ curves using curve fitting techniques (Gu, Pallardy, Tu, Law, & Wullschleger, 2010; Sun, Gu, Dickinson, Pallardy et al., 2014). An alternative approach as applied in this study makes use of independent *g*_m_ estimates which allow the conversion of *A*_n_ − *C*_i_ curves to *A*_n_ − *C*_c_ curves and the subsequent re‐estimation of photosynthetic parameters on a *C*_c_ basis. This alternative approach consists of three main steps (illustrated in Figure S3; R code available at https://bitbucket.org/juergenknauer/mesophyll_conductance):

1. Simulation of a PFT‐specific *A*_n_ − *C*_i_ curve under unstressed conditions, saturating light, and 25°C using the current (implicit *g*_m_) photosynthesis routine of the model with *C*_i_‐based Rubisco parameters from Bernacchi et al. (2001). Under these conditions, *g*_m_ is assumed to equal *g*_m,max25_.
2. Calculation of *C*_c_ from Fick's first law: *C*_c_ = *C*_i_ – *A*_n_/*g*_m_ and construction of the corresponding *A*_n_ − *C*_c_ curve. Depending on whether *g*_m_ is assumed to be independent of *C*_i_ or not, *g*_m_ is either assumed to be constant or a function of *C*_i_ (Equation (5)).
3. Simultaneous fitting of *V*_cmax25_ and *J*_max25_ to the *A*_n_ − *C*_c_ curve calculated in Step 2 using the same model as in step 1, but with *C*_c_‐based Rubisco parameters taken from Bernacchi et al. (2002). The fitting is done with a non‐linear regression routine.

Compared to parameter adjustments based on measured *A*_n_ − *C*_i_ curves, this approach has the advantage of being universally applicable across model types and model structures, and to both C3 and C4 photosynthesis models. This flexibility is achieved by an internally consistent parameter adjustment which is ensured by the employment of the exact same photosynthesis model and parameter values (e.g. leaf day respiration, Rubisco kinetic parameters) for both the parameter adjustment and the actual model simulations. In addition, this approach circumvents uncertainties associated with the determination of *g*_m_ from *A*_n_ − *C*_i_ curves (e.g. assignment of limitation states) by taking independent *g*_m_ measurements. It follows that no raw data (i.e. *A*_n_ − *C*_i_ curves) are required, but instead a sufficient number of *g*_m_ measurements, from which representative estimates of *g*_m_ can be inferred.

### 2.6 Site‐level simulations

The JSBACH model was run for eight eddy covariance sites within the FLUXNET network. The sites were selected to cover different PFTs and contrasting hydro‐climates (Table 2). Meteorological data for all sites was downloaded from the FLUXNET2015 webpage (http://fluxnet.fluxdata.org/data/fluxnet2015-dataset/; accessed 2017‐11‐09).

**Table 2:** Characteristics of eddy covariance sites used in this study

Site	Vegetation type	Simulation period	MAT^a^ (°C)	MAP^b^ (mm)	Max. LAI	Vegetation height (m)	*V*_cmax25,Ci_ (μmol m^−2^ s^−1^)	*g*_m,max25_ c (mol m^−2^ s^−1^)	Site reference
AT‐Neu	C3 grasses/herbs	2008–2012	6.3	852	6	0.5	70	0.197	Wohlfahrt et al. (2008)
DE‐Geb	C3 crops	2005–2014	8.5	470	5	0.5	39	0.295	Kutsch et al. (2010)
FI‐Hyy	Evergreen needle‐leaf forest	1996–2014	3.8	709	3.3	14	41	0.090	Vesala et al. (2005)
FR‐LBr	Evergreen needle‐leaf forest	2003–2008	13.6	900	3.1	18	42	0.090	Berbigier, Bonnefond, and Mellmann (2001)
FR‐Pue	Evergreen broadleaf forest	2005–2014	13.5	883	3.3	5.5	24	0.106	Rambal et al. (2003)
GF‐Guy	Tropical rainforest	2006–2014	25.7	3,041	7	35	36	0.152	Bonal et al. (2008)
US‐Ha1	Deciduous broadleaf forest	1992–2012	6.6	1,071	4.9	23	45	0.176	Urbanski et al. (2007)
US‐Ne1	C4 crops (irrigated maize)	2002–2012	10.1	790	6	3	32	0.739	Verma et al. (2005)

a Mean annual temperature.

b Mean annual precipitation.

c Cover fraction‐weighted *g*_m,max25_ values of the plant functional types present at the site.

All sites were run with meteorological forcing from the flux towers. Vegetation height, roughness length, and LAI were adjusted according to values reported in the literature, and *C*_i_‐based photosynthetic capacity (*V*_cmax25,Ci_ and *J*_max25,Ci_) was adjusted to match the flux measurements. For all sites, all model versions (*Imp*,* Exp*,* ExpC*,* ExpL*,* ExpCL*) were forced with (1) observed meteorological conditions and (2) elevated CO_2_ concentrations (ambient + 200 μmol/mol), and the same meteorological forcing as in the ambient CO_2_‐runs.

### 2.7 Global simulations

To investigate the large‐scale implications of an explicit representation of *g*_m_ in JSBACH, we conducted global runs for the *Imp*,* Exp*, and *ExpCL* model versions under historical (1970–2004) and projected future conditions (2070–2099). Bias‐corrected daily meteorological forcing (0.5° spatial resolution) for both the historical and future runs was obtained from the Inter‐Sectoral Impact Model Intercomparison Project (ISIMIP) (Frieler et al., 2017; Hempel, Frieler, Warszawski, Schewe, & Piontek, 2013), using output from the HadGEM2‐ES model (Martin et al., 2011). Future runs were conducted with RCP4.5 and RCP8.5 scenarios. Land cover was obtained from Pongratz, Reick, Raddatz, and Claussen (2007) and assumed to be unchanged in the historical and future runs. *g*_m,max25_ as well as *V*_cmax25_ and *J*_max25_ values are listed in Table 3.

**Table 3:** *g*_m,max25_, *C*_i_‐based and *C*_c_‐based *V*_cmax25_ and *J*_max25_, and *J*_max25_/*V*_cmax25_ ratios for different plant functional types (PFTs) in the JSBACH model and for the *Exp* and *ExpC* model versions. Adjustments of *C*_i_‐ to *C*_c_‐based parameters were performed as described in Section 2.5. *V*_cmax25,Ci_ values were taken from Kattge et al. (2009), and if applicable re‐calculated based on N_a_ (leaf nitrogen per area) data in Kattge et al. (2011). PFT abbreviations are as in Figure 1

PFT	*g*_m,max25_ ± SEM (mol m^−2^ s^−1^)	*V*_cmax25,Ci_ (μmol m^−2^ s^−1^)	*J*_max25,Ci_ (μmol m^−2^ s^−1^)	*J*_max25,Ci_/*V*_cmax25,Ci_	*V*_cmax25,Cc_ (μmol m^−2^ s^−1^)	*J*_max25,Cc_ (μmol m^−2^ s^−1^)	*J*_max25,Cc_/*V*_cmax25,Cc_	*g*_m,max25_ a (mol m^−2^ s^−1^)	*V*_cmax25,Cc_ (μmol m^−2^ s^−1^)	*J*_max25,Cc_ (μmol m^−2^ s^−1^)	*J*_max25,Cc_/*V*_cmax25,Cc_
	*Exp*	*Imp*	*Imp*	*Imp*	*Exp*	*Exp*	*Exp*	*ExpC*	*ExpC*	*ExpC*	*ExpC*
DNF	0.057 ± 0.008	33.1	62.9	1.9	59.3	65.0	1.10	0.054	68.1	89.9	1.32
TDF	0.058 ± 0.020	31.0	58.9	1.9	49.8	60.4	1.21	0.056	50.4	76.9	1.41
ENF	0.078 ± 0.021	52.7	100.1	1.9	118.8	105.5	0.89	0.074	113.3	145.3	1.28
DSH	0.098 ± 0.025	49.8	94.7	1.9	75.7	96.9	1.28	0.100	78.1	112.1	1.44
EBF	0.101 ± 0.010	61.4	116.7	1.9	117.8	121.1	1.03	0.100	126.7	167.4	1.32
TRF	0.152 ± 0.026	39.0	74.1	1.9	42.1	74.1	1.76	0.151	43.7	76.9	1.76
DBF	0.175 ± 0.016	52.1	98.9	1.9	58.6	99.2	1.69	0.172	61.3	104.2	1.70
C3G	0.197 ± 0.015	50.1	95.2	1.9	54.0	95.1	1.76	0.198	55.8	98.7	1.77
RSH	0.224 ± 0.111	49.8	94.7	1.9	52.1	94.4	1.81	0.230	53.6	97.3	1.82
C3C	0.295 ± 0.017	80.2	152.4	1.9	87.9	152.5	1.73	0.305	90.3	158.5	1.76

		*V*_pmax25,Ci_ (μmol m^−2^ s^−1^)			*V*_pmax25,Cc_ (μmol m^−2^ s^−1^)				*V*_pmax25,Cc_ (μmol m^−2^ s^−1^)		
C4G	0.452 ± 0.151	40.0			118.9			0.382	189.0		
C4C	0.739 ± 0.474	60.0			145.4			0.620	193.6		

SEM: standard error of the median.

a Standardized to a *C*_i_ of 260 μmol mol^−1^ (Equation (5)).

## 3 RESULTS

### 3.1 Unstressed *g*_m_ values across PFTs

Figure 1 shows the results of the literature review, revealing distinct patterns in unstressed *g*_m_ across PFTs. Lowest values were found in needle‐leaf and evergreen broadleaf trees, followed by tropical evergreen trees and deciduous broadleaf trees. Generally, herbaceous species had higher *g*_m_ values than woody species. Within herbaceous PFTs, crops had higher *g*_m_ values than grasses and wild herbs, and C4 plants had higher values than C3 plants. The number of measurements was unequally distributed among the PFTs and 87% of all measurements were performed in only four PFTs (C3C, EBF, C3G, DBF). It follows that most PFTs are poorly sampled and the corresponding *g*_m_ measurements are less robust than in the well‐sampled PFTs. However, the four highly sampled PFTs also showed a large spread, reflecting the wide range of *g*_m_ values among measurement methods or among different species within each PFT. Results in Figure 1 show *g*_m_ values that were not standardized to a given *C*_i_ or to high light. Accounting for a potential response of *g*_m_ to light or *C*_i_ led to only minor changes in the magnitude of *g*_m_ and its pattern across PFTs (Table 3 and Table S2).

### 3.2 Parameter adjustment

The required parameter adjustment procedure as described in Section 2.5 led to significant changes to the two key photosynthetic parameters in the model, *V*_cmax25_ and *J*_max25_ (Table 3). The *C*_c_‐based parameters (*V*_cmax25,Cc_ and *J*_max25,Cc_) account for the lower available CO_2_ concentration due to the effects of *g*_m_, and are thus usually higher than their *C*_i_‐based counterparts. For all PFTs, *V*_cmax25_ was more strongly affected than *J*_max25_, which resulted in a decrease of the *J*_max25_/*V*_cmax25_ ratio. The difference between the *C*_i_‐based and *C*_c_‐based parameters depends both on the magnitude of *g*_m_ and the magnitude of *V*_cmax25,Ci_ and *J*_max25,Ci_, and is highest when *g*_m_ is low and photosynthetic capacity is high (as e.g. in ENF). Thus, effects are strongest when the CO_2_ drawdown from the intercellular airspaces to the chloroplasts (*C*_i_ − *C*_c_) is high. The decrease of the *J*_max25,Cc_/*V*_cmax25,Cc_ ratio led to a shift of the inflection point (the *C*_i_ where photosynthetic limitation changes from Rubisco‐limited to RuBP (ribulose‐1,5‐bisphosphate)‐limited) to lower *C*_i_, which is associated with a higher fraction of photosynthesis occurring in the electron transport‐limited domain (see e.g. Figure S3).

In the model versions where *g*_m_ is affected by *C*_i_ (*ExpC* and *ExpCL*), *g*_m_ was assumed to change throughout the *A*_n_ − *C*_i_ curve according to Equation (5), that means it increases sharply at low *C*_i_ and decreases continuously thereafter. When accounting for this potential response, the re‐adjusted photosynthetic parameters, in particular *J*_max25,Cc_, were considerably higher compared to the default version (*Exp*). The higher *J*_max25,Cc_ compensates for the low *g*_m_ simulated at higher *C*_i_ where RuBP‐regeneration is limiting (Figure S1), and thus maintains the same *A*_n_ at high *C*_i_ as in the implicit case. *ExpC* and *ExpCL* were thus characterized by significantly higher *J*_max25,Cc_/*V*_cmax25,Cc_ ratios compared to the *Exp* model version (Table 3). The model versions accounting for a light response of *g*_m_ (*ExpL* and *ExpCL*) did generally not show strong deviations from the corresponding versions without a light response (*Exp* and *ExpC*, respectively), but tended to have lower *V*_cmax25,Cc_ values and slightly higher *J*_max25,Cc_/*V*_cmax25,Cc_ ratios (Table S2).

In the C4 photosynthesis model described by von Caemmerer and Furbank (1999), the only parameter affected by the parameter adjustment is the maximum PEP‐carboxylation rate (*V*_pmax25_) (Figure S4). In case of a *g*_m,max25_ of 0.739 mol m^−2^ s^−1^, the median value observed in C4 crops, *V*_pmax25_ increased strongly from 60 (*C*_i_‐based) to approximately 145 μmol m^−2^ s^−1^ (*C*_m_‐based; Table 3).

### 3.3 Effects on simulated leaf‐level photosynthesis

**Figure 2:**
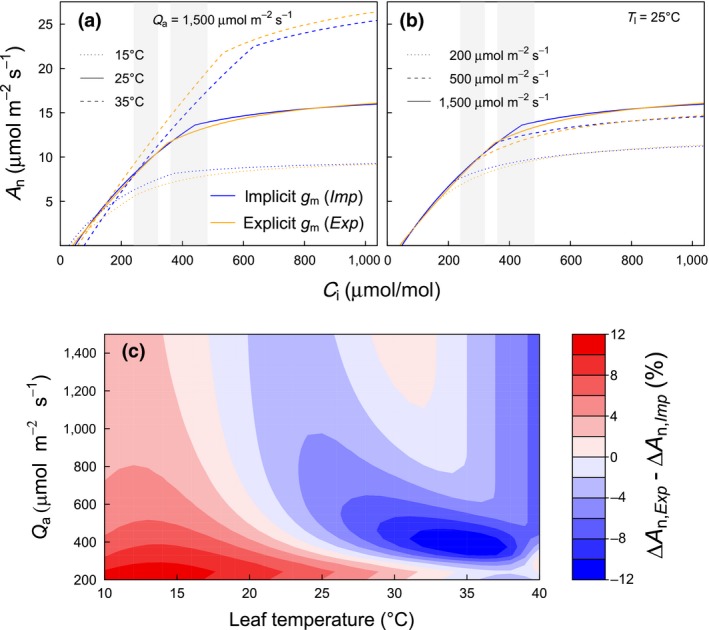
(a) *A*_n_ − *C*_i_ curves for the implicit (*Imp*, blue) and explicit (*Exp*, orange) model versions for three different temperatures (b) and light conditions and (c) the resulting differences in photosynthetic sensitivity to CO_2_ between the implicit and the explicit model version for the grey shaded *C*_i_ regions in (a) and (b). Δ*A*_n_ in (c) is defined as Δ*A*_n_ = (*A*_n,eCO2_ − *A*_n,aCO2_)/*A*_n,aCO2_ × 100, where aCO_2_ denotes the intercellular CO_2_ concentration (*C*_i_) range between 240 and 320 μmol/mol (=0.6 × 400 – 0.8 × 400 μmol/mol) and eCO_2_ denotes the *C*_i_ range between 360 and 480 μmol/mol (=0.6 × 600 – 0.8 × 600 μmol/mol). Positive values in (c) indicate that *A*_n_ in the explicit model is more sensitive to CO_2_ than *A*_n_ in the implicit model, negative values indicate the opposite. Shown are leaf‐level simulations using the C3 photosynthesis model described in Farquhar et al. (1980) with the following parameters: *V*_cmax25,Ci_ = 40 μmol m^−2^ s^−1^; *J*_max25,Ci_ = 76 μmol m^−2^ s^−1^; *g*_m,max25_ = 0.1 mol m^−2^ s^−1^; *V*_cmax25,Cc_ = 50.8 μmol m^−2^ s^−1^; *J*_max25,Cc_ = 76.86 μmol m^−2^ s^−1^; *R*_l_ (respiration rate in light) = 0.44 μmol m^−2^ s^−1^; and Rubisco kinetic parameters as listed in Table S1

Simulated photosynthesis in the implicit (*Imp*) and the explicit (*Exp*) model versions are compared in Figure 2. Shown are *A*_n_ − *C*_i_ curves calculated from leaf‐level simulations under contrasting temperature and light conditions. The adjustment of *V*_cmax25_ and *J*_max25_ was always performed under reference conditions (i.e. temperature of 25°C and saturating light) and aimed to minimize the difference between the implicit and explicit simulations under these reference conditions (Figure 2a, solid lines). The achieved goodness of fit depends on the magnitude of *g*_m_, with lower *g*_m_ resulting in a poorer fit to the implicit *A*_n_ − *C*_i_ curve under otherwise equal conditions (Table S3). Importantly, when temperature and light deviate from the reference conditions, the agreement between the implicit and explicit model deteriorates. This is especially relevant when temperature changes, because *g*_m_ exhibits a strong temperature response (Equation (3)), leading to higher and lower *A*_n_ at temperatures higher and lower than 25°C, respectively (Figure 2a). The model comparison at lower light conditions (Figure 2b) did not necessarily lead to a poorer agreement between the model versions, but the comparison exemplifies that the mismatch between the model versions and thus the sensitivities to CO_2_ strongly depends on the prevailing conditions. The *ExpC* model led to similar curves as shown in Figure 2 (Figure S6). Assuming that *g*_m_ responds to light (*ExpL*) led to much lower simulations of *A*_n_ under low light, as well as to higher sensitivities to rising CO_2_ throughout the whole *C*_i_ range (Figure S7). The deviations between the implicit and explicit model versions caused changes in the relative sensitivity of *A*_n_ to changes in *C*_i_ compared to the reference conditions (Figure 2c). In general, *A*_n_ showed a stronger relative response to *C*_i_ in the explicit compared to the implicit model at lower temperatures, but the opposite behavior at high temperatures. Note that Figure 2c depicts changes in the sensitivity of *A*_n_ to *C*_i_ in relative terms (see Figure caption), which was decreased at high temperatures despite similar slopes of *A*_n_ − *C*_i_ in Figure 2a. These contrasts were more pronounced at lower light conditions. It has to be noted that the sensitivities and their relation between the implicit and explicit model version depend on the *C*_i_ range of interest (shaded areas in Figure 2a,b), which explains the fact that the CO_2_ effect of *g*_m_ as shown in Figure 2c becomes negative already at temperatures lower than 25°C at high *Q*_a_.

### 3.4 Site‐level simulations

**Figure 3:**
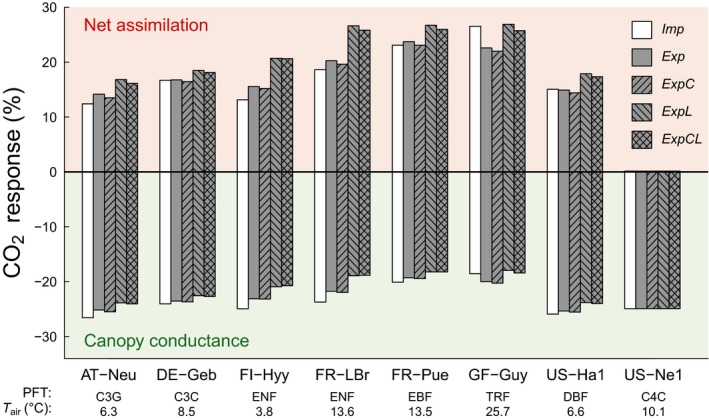
Relative responses of ecosystem‐level net assimilation and canopy conductance to elevated atmospheric CO_2_ concentrations for the five main model versions tested in this study (*Imp* = implicit *g*_m_; *Exp* = explicit *g*_m_; *C* = *C*_i_ response of *g*_m_; *L* = light response of *g*_m_). CO_2_ response is calculated as (X_eCO2_ – X_aCO2_)/X_aCO2_ × 100, where X denotes either canopy net assimilation or canopy conductance, and aCO_2_ and eCO_2_ denote ambient and elevated (ambient + 200 μmol/mol) atmospheric CO_2_ concentrations, respectively. PFT abbreviations are as in Figure 1. T_air_ represents the mean annual temperature (see also Table 2)

The integrated response of ecosystem‐level *A*_n_ (*A*_n,canopy_) and *g*_s_ (canopy conductance, *G*_c_) to changes in atmospheric CO_2_ concentrations are analyzed in Figure 3 for an exemplary set of ecosystems from the FLUXNET2015 database. In the implicit model version (*Imp*), *A*_n,canopy_ increased under eCO_2_ at all sites with C3 vegetation. The relative increases depend on temperature as described previously (Kirschbaum, 1994), and were more pronounced in warm (e.g. GF‐Guy) than in cold climates (e.g. FI‐Hyy). The explicit model version that does not consider a light and *C*_i_ response (*Exp*) showed higher sensitivities of *A*_n,canopy_ to eCO_2_ for most sites, but a significantly lower sensitivity for GF‐Guy, which can be explained by the lower photosynthetic sensitivity to CO_2_ at higher temperatures (Figure 2). The model configuration that responds to *C*_i_, but not to *Q*_a_ (*ExpC*) showed similar or slightly lower responses compared to the *Exp* model version, which is likely due to the fact that the lower simulated *g*_m_ was largely compensated by the higher *C*_c_‐based photosynthetic capacity (Table 3). In contrast, model configurations that simulate a response of *g*_m_ to light (*ExpL* and *ExpCL*) showed the highest responsiveness of *A*_n,canopy_ to CO_2_, which is a consequence of the continuously higher sensitivity under low light in the *ExpL* and *ExpCL* versions due to the marked decrease of *g*_m_ at low light (Equation (6), Figure S7). This effect is amplified at the canopy level, as a considerable fraction of a closed canopy continuously operates at low light conditions.

The positive responses in *A*_n,canopy_ were accompanied by negative responses in *G*_c_, that is stomatal closure. A consistent pattern in Figure 3 is the opposite response of *G*_c_ compared to *A*_n,canopy_ in the sense that a stronger response of *A*_n,canopy_ was associated with a weaker response of *G*_c_, with the result that the response of ecosystem‐level intrinsic water‐use efficiency (iWUE_,canopy_ = *A*_n,canopy_/*G*_c_) to eCO_2_ did not vary among the model runs (i.e. it always increased by the same amount). This can be explained as an intrinsic property of the stomatal model employed here (Medlyn et al., 2011), in which *C*_i_/*C*_a_ is assumed to stay constant with rising CO_2_ concentrations. This model behavior is unchanged in the explicit model version with the consequence that stronger positive responses of *A*_n_ to eCO_2_ are accompanied by weaker decreases in *g*_s_, the combination of which keeps *C*_i_/*C*_a_ constant. Hence, the changes in *g*_s_ are not direct effects of *g*_m_, but indirect ones via *A*_n_ that are caused by the coupling between *A*_n_ and *g*_s_ in the model. This relationship holds regardless of whether *g*_m_ is assumed to stay constant or to decrease over time, as it is the case in the *ExpC* model runs.

For C4 plants, none of the explicit model versions led to any changes in simulations of *A*_n,canopy_ and *G*_c_ compared to the implicit model. All model runs did not show a response of *A*_n,canopy_ to eCO_2_, and a constant decrease of c. 25% in *G*_c_. The reason for this is that *C*_i_ did not fall in the range where photosynthesis is limited by *V*_pmax_ (e.g. low *C*_i_). This behavior depends on the parameterization of the model, and *g*_m_ effects might be more important under conditions of water stress.

**Figure 4:**
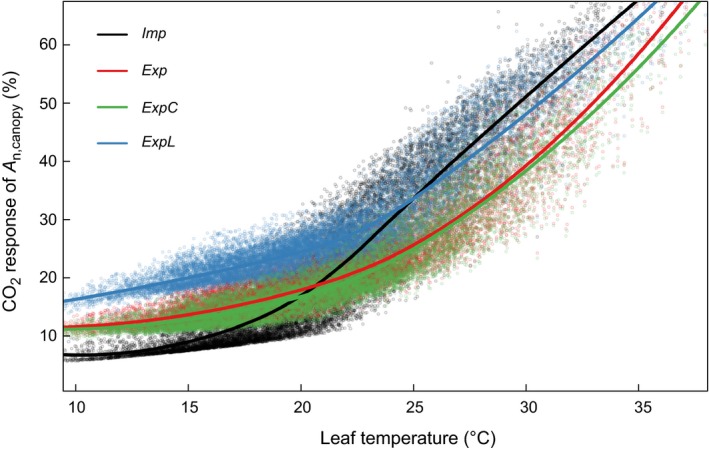
Sensitivity of canopy‐level net assimilation (*A*_n,canopy_) to elevated CO_2_ concentrations in the implicit (*Imp*) and explicit model versions (*Exp*,* ExpC*,* ExpL*) for the Mediterranean pine forest site FR‐LBr. CO_2_ response of *A*_n,canopy_ is defined as (*A*_n,canopy,eCO2_ – *A*_n,canopy,aCO2_)/*A*_n,canopy,aCO2_ × 100, where aCO_2_ and eCO_2_ denote ambient and elevated (ambient + 200 μmol/mol) atmospheric CO_2_ concentrations, respectively. Data were filtered to represent periods in the growing season (four most productive months), at daytime (*Q*_a_ > 200 μmol m^−2^ s^−1^), and in the absence of soil water stress (β > 0.95; Equation (4)). Points are half‐hourly simulation results, and lines indicate local polynomial regression fits (loess) to the points

As shown in Figure 2, the fact that *g*_m_ responds to temperature leads to a significantly different temperature response of *A*_n,canopy_. It follows that the photosynthetic sensitivity to CO_2_ shows a different response to temperature in the explicit compared to the implicit model versions (Figure 4). The sensitivity of *A*_n,canopy_ to CO_2_ increased with temperature in all model versions, but with a different functional response (i.e. slope). In particular at low temperatures (<20°C), the explicit model versions simulated a higher photosynthetic sensitivity to CO_2_ compared to the implicit version. This behavior was reversed at approx. 20°C, above which *Exp* and *ExpC* simulated a lower photosynthetic sensitivity to CO_2_ compared to *Imp*. The *ExpL* version showed the highest sensitivity at low temperatures due to the above mentioned amplification of the light response at canopy level. At high temperatures (above c. 25°C), *ExpL* and *Imp* showed similar temperature sensitivities.

**Figure 5:**
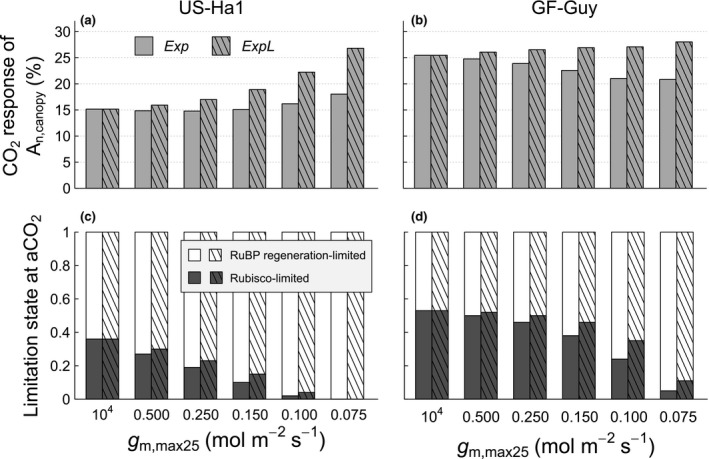
Site‐level simulations for the sites US‐Ha1 and GF‐Guy with differing values of *g*_m,max25_. (a–b) CO_2_ response of *A*_n,canopy_ (defined as in Figure 3). (c–d) Fraction of canopy level net assimilation (*A*_n,canopy_) limited by the two limitation states of the photosynthesis model in the ambient CO_2_ (aCO_2_) simulations. *V*_cmax25_ and *J*_max25_ were re‐adjusted for each *g*_m,max25_ value as described in Section 2.5

As demonstrated in Figures 2, 3, 4, the effects of *g*_m_ on the photosynthetic responses to eCO_2_ depend not only on the magnitude of *g*_m_ (and thus PFT) but also on the environmental conditions, foremost temperature and radiation. To investigate the isolated effects of *g*_m_ without any confounding meteorological factors, we conducted additional ecosystem‐level simulations for the sites US‐Ha1 and GF‐Guy, in which *g*_m,max25_ was varied while keeping the climate forcing unchanged. For these simulations, *g*_m,max25_ was reduced stepwise from 10,000 (i.e. non‐limiting) to 0.075 mol m^−2^ s^−1^, and *V*_cmax25_ and *J*_max25_ were re‐adjusted for each change in *g*_m,max25_ as described in Section 2.5. The results demonstrate that the effects of *g*_m_ on simulations of photosynthesis strengthen when its magnitude decreases (Figure 5). This is a consequence of the increasing mismatch between the implicit and explicit model versions when *g*_m_ decreases (Table S3), an effect that amplifies when conditions deviate from those that were used for the parameter adjustment (Figure 2). In the *Exp* model version, the high temperatures in the tropical site GF‐Guy thus caused the photosynthetic sensitivity to CO_2_ to decrease when *g*_m_ decreases, whereas the opposite was the case in the temperate site US‐Ha1. The *ExpL* version caused a stronger sensitivity with decreasing *g*_m_ for all sites. At US‐Ha1, this led to a significant increase of the photosynthetic sensitivity to CO_2_ at low *g*_m_. At GF‐Guy, in contrast, a potential light response of *g*_m_ offsets the increase caused by a reduced *g*_m_, with the consequence that the *ExpL* model version showed a similar sensitivity for all *g*_m_ values.

At both sites, the proportion of Rubisco‐limited *A*_n,canopy_ decreased when *g*_m,max25_ decreased. Again, this is a consequence of the parameter adjustment (see Section 2.5), in which the stronger changes in *V*_cmax25_ compared to *J*_max25_ lead to a shift of the inflection point to a lower *C*_i_, which is associated with a higher fraction of photosynthesis occurring in the RuBP regeneration‐limited domain. This effect (i.e. the change in *J*_max25_/*V*_cmax25_) increases with a decrease in *g*_m_ under otherwise equal conditions. In general, this shift towards lower proportions of Rubisco‐limited photosynthesis on total canopy level photosynthesis counteracts the higher photosynthetic sensitivity to CO_2_ caused by an explicit *g*_m_, as photosynthesis in the RuBP regeneration limited region shows a lower sensitivity to rising CO_2_ concentrations.

### 3.5 Global simulations

**Figure 6:**
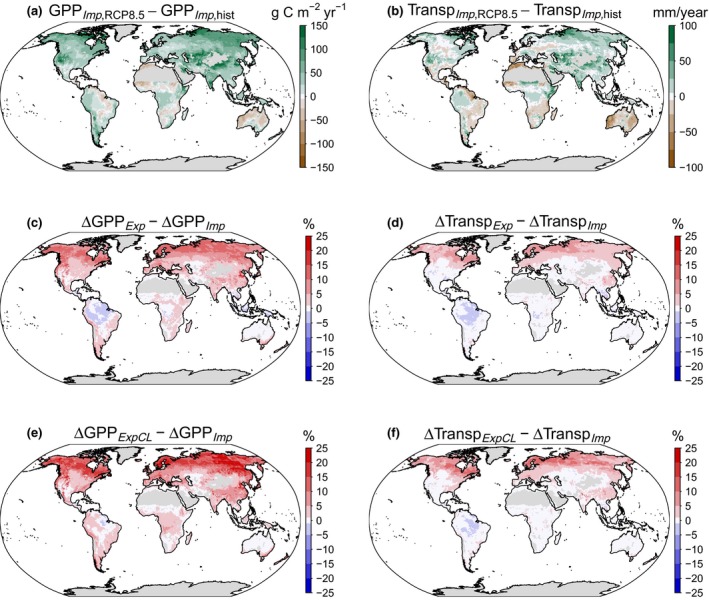
(a–b) Simulated differences between the RCP8.5 future scenario (2070–2099) and the historical (hist) runs (1975–2004) in mean annual gross primary productivity (GPP) and Transpiration (Transp) in the implicit (*Imp*) model version. (c–f) Relative differences between the *Imp* and the *Exp* (c–d) and *ExpCL* (e–f) model versions. Δ is defined as Δ = (X_RCP8.5_ − X_hist_)/X_hist_ × 100, where X is either GPP or Transpiration. Regions with an average annual GPP < 200 g C m^−2^ yr^−1^ were masked out. Red colors in panels c–f denote stronger increases in GPP or Transpiration in the *g*_m_‐explicit simulations compared to the *g*_m_‐implicit simulations

At the global scale, the widespread substantial increases in mean annual GPP from the historical (1975–2004) to the future RCP8.5 (2070–2099) simulation illustrate the commonly observed CO_2_‐fertilization effect (Figure 6a). Exceptions from this upward trend were found in some semi‐arid regions, as well as in parts of the Amazon basin, which experience a drying trend in the climate projections by HadGEM2‐ES. Transpiration (Figure 6b), showed weaker absolute responses and a more diverse pattern throughout the globe. In contrast to GPP, transpiration tended to be reduced due to stomatal closure, but this reduction may be offset by increasing VPD in some regions of the earth (Kala et al., 2016). The more moderate RCP4.5 future scenario showed similar patterns, but smaller absolute differences (Figure S8). Figure 6 further reveals that *g*_m_ had spatially contrasting effects on the photosynthetic sensitivity to CO_2_. The differences in the ∆ values between the *Exp* and the *Imp* version largely reflect both the magnitude of *g*_m_ (and thus vegetation type), and the environmental conditions as described earlier. It follows that the largest changes could be found in high latitudes, in particular in boreal forests, which show a combination of vegetation with a low *g*_m_ (ENF and DNF) and a cool climate, both of which increased the photosynthetic sensitivity to CO_2_ when *g*_m_ is modeled explicitly. Changes were moderately positive throughout large parts of the temperate (+5% to +15%) and semi‐arid regions of the earth (+0% to +5%) and slightly negative in large parts of the inner tropics (−2% to 0%). This decrease in the CO_2_ sensitivity of photosynthesis is in accordance with the site‐level results, and is mostly attributable to the high temperatures in these regions (Figure 2). The *ExpCL* model version (Figure 6e,f) showed similar spatial patterns as the *Exp* model, but consistently stronger positive responses. The reason for the stronger response is the light response function that amplifies at canopy level, as described earlier. The changes in transpiration in both the *Exp* and *ExpCL* model versions mirror those found for GPP, but are generally weaker. The weaker responses of transpiration compared to GPP are likely caused by aerodynamic decoupling, that cause a lower sensitivity of modeled transpiration to atmospheric CO_2_ compared to *G*_c_ (Knauer et al., 2017).

**Figure 7:**
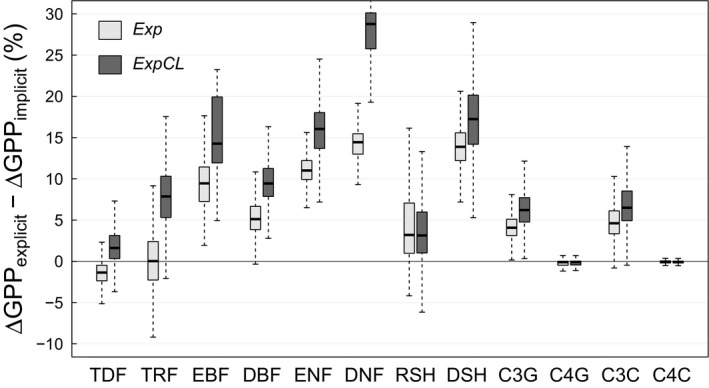
Photosynthetic sensitivity to future climate conditions for different plant functional types (PFTs), expressed as the differences between the ∆GPP of the explicit (*Exp* or *ExpCL*) and implicit (*Imp*) *g*_m_ model versions. ∆GPP was calculated as (∆GPP = GPP_RCP8.5_ – GPP_hist_)/GPP_hist_ × 100, where GPP_RCP8.5_ and GPP_hist_ denote GPP simulated in the RCP8.5 future scenario (2070–2099) and the historical runs (1975–2004), respectively. Shown are results at tile‐level (i.e. GPP is simulated only for the respective PFT and not for the entire grid cell) and only for grid cells where the cover fraction of the respective PFT was at least 30%. PFT abbreviations are as in Figure 1

The differences among plant types are more clearly demonstrated in Figure 7 (for the RCP8.5 scenario; see Figure S9 for the RCP4.5 scenario). As stated earlier, the differences among the PFTs are not only caused by differences in *g*_m_, but also by differences in the prevailing climatic conditions. For example, the lower response of TDF compared to DNF, despite similar *g*_m,max25_ values, can be attributed to the higher temperatures the TDF are exposed to (Figure 4). Nonetheless, the comparison of co‐occurring PFTs in the same model grid cells (i.e. PFTs experiencing identical climate forcing), showed significant differences in the simulated photosynthetic sensitivity, indicating that changes therein can primarily be attributed to differences in *g*_m_, and not to climate (Figure S10).

The widespread increases in plant carbon uptake in the explicit model versions relative to the implicit version of 5%–25% between 1975–2004 and 2070–2099 (Figures 6 and 7) are reflected in the clear increases in simulations of global GPP (Figures S11–S13). Differences in the simulated global GPP values in the RCP8.5 scenario between the *Imp* and the *Exp* and *ExpCL* model versions amounted to 3.6 and 6.6 Pg C yr^−1^, respectively, for the 2070–2099 period. In both cases, about two‐third of the increase was caused by regions north of 30°N (Figure S11), in particular in boreal forests (Figure 7).

## 4 DISCUSSION

### 4.1 Required adjustments to the Farquhar et al. (1980) photosynthesis model

The explicit consideration of *g*_m_ in models of photosynthesis requires that photosynthetic parameters are adjusted from their apparent (*C*_i_‐based) to true (*C*_c_‐based) values, as the former implicitly account for the effects of *g*_m_. The Rubisco kinetic parameters (*K*_o_, *K*_c_ and Γ*) as well as their activation energies have been determined by for exmaple Bernacchi et al. (2002) on a *C*_c_ basis. These parameters are commonly assumed to be conserved across C3 plants (but see e.g. Walker et al., 2013), which leaves the species‐specific parameters *V*_cmax25_ and *J*_max25_ left to adjust. Here, we suggest a simple and flexible parameter adjustment scheme that is applicable across model representations of photosynthesis (Figures S3–S5) and that does not require measured *A*_n_ − *C*_i_ curves, but instead independent *g*_m_ estimates. The approach ensures that *V*_cmax25_ and *J*_max25_ are converted in accordance with the individual structure and parameterization of the photosynthesis model. This consistency of *V*_cmax25,Cc_ and *J*_max25,Cc_ with the other parameters in the model (e.g. Rubisco kinetics) could not be assured if *V*_cmax25,Cc_ and *J*_max25,Cc_ were taken directly from leaf‐level measurements, as these values are often derived assuming different photosynthetic parameters than the model (see Table S4 for a sensitivity analysis). It should be noted that the original *C*_i_‐based estimates of *V*_cmax_ and *J*_max_ might not represent *A*_n_ − *C*_i_ curves well due to the assumption of an infinite *g*_m_ (Ethier & Livingston, 2004). Any potential bias inherent in the *C*_i_‐based parameters will be propagated to their *C*_c_‐based values (Table 3). However, the degree of bias in the *C*_i_‐based parameters as well as the actual implications for the derived *C*_c_‐based parameters still needs to be investigated.

The adjustment from apparent to true values resulted in changes to the key parameters *V*_cmax25_ and *J*_max25_ that are qualitatively comparable to the results of previous adjustments (Sun, Gu, Dickinson, Pallardy et al., 2014), and that compare well with independently adjusted parameters by Bahar, Hayes, Scafaro, Atkin, and Evans (2018) (results not shown). The adjustment again underlines the asymmetrical effects that *g*_m_ has on *V*_cmax25_ and *J*_max25_. The stronger change in *V*_cmax25_ compared to *J*_max25_ as a result of the re‐adjustment decreases the *J*_max25_/*V*_cmax25_ ratio and shifts the inflection point towards lower *C*_i_ values. In general, these changes to the photosynthesis model result in an altered response of photosynthesis to key environmental factors like temperature and light. Further, it also changes the sensitivity of photosynthesis to eCO_2_ in dependence on the environmental conditions. This can mostly be attributed to the fact that the parameter adjustment is performed under reference conditions of 25°C and saturating light. Under these conditions, the agreement between the explicit and implicit model versions is the best, but it deteriorates when conditions deviate from the reference conditions, an effect that was previously asserted by Sun, Gu, Dickinson, Norby et al. (2014). Most relevant in this context is the strong temperature response of *g*_m_ (Equation (3)), which leads to a significant deviation of simulated photosynthesis under higher and lower temperatures in the explicit model version. It may be noted that these introduced deviations could be avoided by additionally re‐adjusting the activation energy of *V*_cmax,Cc_. This would, however, not be in accordance with the adjustment of the Rubisco kinetic parameters as performed in Bernacchi et al. (2002), where changes in the temperature response of *A*_n_ were entirely attributed to *K*_c_ and *K*_o_, thereby assuming an unchanged activation energy of *V*_cmax_. This approach is also justified theoretically since *V*_cmax,Cc_, the substrate‐saturated photosynthesis rate, is by definition unaffected by *g*_m_. We thus argue that the observed changes in the photosynthesis response to temperature are not an artifact.

In this study (as in many others), the assumption was made that Rubisco kinetic parameters as determined in tobacco (i.e. following Bernacchi et al., 2002) adequately represent all PFTs. Recent studies have found notable differences in Rubisco kinetic parameters across plant types and species (Galmés, Hermida‐Carrera, Laanisto, & Niinemets, 2016; Walker et al., 2013), and differences in Rubisco properties across plant types and climate conditions, as outlined in Galmés et al. (2016), should be included in future LSMs to better represent the temperature response of photosynthesis across the globe. However, so far no studies have determined Rubisco kinetic parameters on a *C*_c_‐basis across PFTs, which could be used in LSMs where *g*_m_ is included explicitly. For use in models, it is essential that *C*_c_‐based Rubisco kinetic parameters, *g*_m_, as well as their temperature responses, are measured on the same set of leaves (as in Bernacchi et al., 2002), in order to ensure consistency across photosynthetic parameters.

### 4.2 Implications for water and carbon fluxes at ecosystem level

The adjustments to the photosynthesis model cause modest changes to the CO_2_ sensitivity of *A*_n,canopy_ and *G*_c_. However, the responses depend on the type of *g*_m_ model that is implemented. In the *Exp* version (no light and *C*_i_ response), the sensitivity of *A*_n,canopy_ and *G*_c_ to CO_2_ depends both on the magnitude of *g*_m_ and the climatic conditions, foremost temperature. Strongest effects were found in cold ecosystems with a low *g*_m_ (FI‐Hyy), but this response does not hold across all climate types, and the tropical site GF‐Guy showed the reverse response and a decreasing responsiveness to eCO_2_.

The *ExpC* version (*C*_i_ response) does not differ markedly from the *Exp* version described above for any of the ecosystems investigated here. This indicates that the parameter adjustment is capable of completely offsetting the *g*_m_ response to *C*_i_ by a concomitant increase in *J*_max25,Cc_. This is an important implication for models as our results indicate that a potential response of *g*_m_ to *C*_i_ is not expected to have an impact on the simulated response of carbon and water fluxes to eCO_2_ in LSMs.

Contrarily, the *ExpL* version (light response) leads to a stronger CO_2_ responsiveness of *A*_n,canopy_ in all ecosystem types. This effect can best be demonstrated with leaf level simulations under low light conditions, where the CO_2_ responsiveness of *A*_n,canopy_ is higher in the *ExpL* compared to the *Imp* model, so long as *C*_i_ is not saturating (Figure S7). This effect is amplified at the ecosystem level, where a certain fraction of the canopy operates in sub‐saturating light conditions regardless of the amount of incident radiation. This potential light response of *g*_m_ thus significantly increases the CO_2_ sensitivity of all C3 ecosystems investigated here and amplifies the strong positive changes in photosynthetic responsiveness to CO_2_ in cold climates, or compensates for the negative response in warmer climates.

The explicit representation of *g*_m_ did not change simulations of iWUE_,canopy_ (*A*_n,canopy_/*G*_c_), which increased at the same rate as in the implicit model version regardless of the *g*_m_ model employed. This behavior is a consequence of the implemented stomatal conductance model (Medlyn et al., 2011), which is based on the strong coupling between *A*_n_ and *g*_s_, that causes the *C*_i_/*C*_a_ ratio to stay constant regardless of the atmospheric CO_2_ concentration. Since most LSMs employ similar *g*_s_ models as the one used here (see e.g. Sato, To, Takahashi, & Katul, 2015 for an overview), our results are in that respect likely representative for most LSMs.

Our results do not indicate changes to simulations of C4 photosynthesis when *g*_m_ is considered explicitly. This is because the explicit consideration of *g*_m_ was compensated by an increase in the PEP‐carboxylation rate (*V*_pmax25_) in the course of the parameter adjustment. While we acknowledge that we lack sufficient data to confidently parameterize the C4 photosynthesis model employed here (von Caemmerer & Furbank, 1999) at the global scale, we argue that, from a modeling point of view, results would be similar if the simpler and more widely used model by Collatz, Ribas‐Carbo, and Berry (1992) (as described in e.g. Bonan et al., 2011) was used, in which case the consideration of *g*_m_ would affect the slope of the initial CO_2_ response curve in a similar manner as it affected *V*_pmax25_ in the model of von Caemmerer and Furbank (1999) (see Figure S5).

### 4.3 Global implications

Global simulations under anticipated future climate suggest clear and regionally contrasting effects of *g*_m_ on GPP and transpiration. The differences between the *g*_m_‐implicit and *g*_m_‐explicit simulations depend on the projected climate, and on the PFT distribution through vegetation‐type differences in the magnitude of *g*_m_. The fact that plant groups with a low *g*_m_ showed stronger responses to eCO_2_ than those with a high *g*_m_ under the same climate generally supports an earlier hypothesis that evergreen species are more likely to have a competitive advantage over other plant types in a high CO_2_ world (Niinemets et al., 2011). However, our analysis suggests that this hypothesis does not hold in the tropics, where a low *g*_m_ led to a decrease in the photosynthetic CO_2_ responsiveness (Figures 5b and 6). However, the actual relevance of *g*_m_ in present and future vegetation dynamics must still be investigated using experimental and modeling approaches.

The replacement of the *g*_m_‐implicit with a *g*_m_‐explicit model caused significant changes to simulations of GPP, ranging from 2.3 Pg C yr^−1^ in the *Exp* model and the RCP4.5 scenario to 6.6 Pg C yr^−1^ in the *ExpCL* model and the RCP8.5 scenario (Figure S11). About two thirds of this increase were caused by regions north of >30°N, where it mostly occurred in regions covered by boreal forests. Changes of this magnitude are likely large enough to significantly affect the amplitude of atmospheric CO_2_ in the high latitudes, hence *g*_m_, which is so far neglected in this context (Forkel et al., 2016; Zeng et al., 2014), should be considered as an additional explanatory factor.

Although our results are broadly consistent with those of Sun, Gu, Dickinson, Norby et al. (2014), our estimates of the GPP response to CO_2_ are more moderate. Compared to the 16% increase in the cumulative GPP found by Sun, Gu, Dickinson, Norby et al. (2014), our results (calculated from Figure S13 using Equation (2) in Sun, Gu, Dickinson, Norby et al., 2014) suggest smaller changes in the order of 6% in the *Exp* model version and the RCP8.5 scenario (but similar values of 15% in the *ExpCL* scenario). However, these numbers may not be directly comparable due to different simulation periods. With respect to the latitudinal patterns of the *g*_m_ effects, our results agree with those by Sun, Gu, Dickinson, Norby et al. (2014), as in both cases, the weakest and strongest effects were found in the tropics and the northern latitudes, respectively. Our simulations (Figure S12) further suggest clear differences in ET between the two model versions, which may have impacts on other physical land surface properties, such as land surface temperature, soil moisture, or sensible heat fluxes.

### 4.4 Future model developments and research needs

Our results emphasize that the absolute value of *g*_m_ is important for the adjustment of photosynthetic parameters and the associated effects on simulations of photosynthesis. The magnitude of *g*_m_ is relatively robust for well‐sampled PFTs, and similar to the results of earlier data compilations (Flexas et al., 2008), but more measurements are needed for tropical species, deciduous needle‐leaf species, and C4 plants. The parameterization of these plant types is important for large‐scale simulations, but their maximum *g*_m_ values can at the moment not be confidently parameterized due to a lack of data. In addition, other plant groups such as ferns should be investigated in future LSMs. These plant groups are characterized by a low *g*_m_ (Carriquí et al., 2015; Tosens et al., 2016), thus their consideration may have important effects on simulated water and carbon fluxes in models that explicitly simulate *g*_m_.

It is clearly desirable to bring empirical formulations of *g*_m_ as used here and in previous studies (Suits et al., 2005; Sun, Gu, Dickinson, Norby et al., 2014) to a more process‐based representation. While existing leaf‐level models of *g*_m_ (Tholen & Zhu, 2011; Tomás et al., 2013) are likely too complex to be parameterized at large scales, global models of *g*_m_ could be readily improved by relating key model parameters (e.g. *g*_m,max25_) to both anatomical (e.g. cell wall thickness, mesophyll porosity) (Peguero‐Pina et al., 2017; Tomás et al., 2013), and biochemical plant traits (e.g. leaf nitrogen content) (von Caemmerer & Evans, 1991; Xue, Ko, Werner, & Tenhunen, 2017; Yamori, Nagai, & Makino, 2011) within a parsimonious model framework that can be applied across plant types.

Currently, one factor hampering future model development is the poor process understanding of *g*_m_, which is associated with the fact that measurements of *g*_m_ are challenging (Pons et al., 2009). It is particularly critical that the role of environmental factors such as *C*_i_ and light is unresolved. Here, we tested the potential effects of these two drivers on large‐scale simulations of carbon and water fluxes. We found that a potential *C*_i_ response does not change model predictions, as its effects would be offset by the adjustment of *V*_cmax25_ and *J*_max25_ from their apparent to true values. A potential light response of *g*_m_, however, would be amplified at canopy level and lead to a significantly higher responsiveness of *A*_n_ to rising atmospheric CO_2_ concentrations. Extrapolated to the global scale, such a leaf‐level response would significantly increase global carbon uptake and water loss. It is thus highly relevant that potential measurement artifacts are ruled out (Gu & Sun, 2014), and that the recently put forward hypothesis of an apparent light response (Théroux‐Rancourt & Gilbert, 2017) is investigated further, as its existence would mean that the light response of *g*_m_ as observed at the leaf level should not be implemented in models.
