# Supplementary material for: Effects of mesophyll conductance on vegetation responses to elevated CO2 concentrations in a land surface model
Source: Glob Chang Biol. 2019 Mar 23;25(5):1820–38. doi: 10.1111/gcb.14604 (PMC6487956; doi:10.1111/gcb.14604)
Supplement: Supplementary file 1 [file GCB-25-1820-s001.docx]

**Appendix S1: Calculation of net assimilation in the JSBACH model**

In both the *g*_m_-implicit (*Imp*) and the *g*_m_-explicit model versions (*Exp, ExpC, ExpL, ExpCL*), net CO_2_ assimilation (*A*_n_) is given by the lesser of the Rubisco-limited net assimilation rate (*A*_c_), and the RuBP regeneration-limited net assimilation rate (*A*_j_) as described in Farquhar *et al.* (1980):

|  | $A_{n}=\min\left( A_{c},A_{j} \right)$ | (S1) |
| --- | --- | --- |

In the *Imp* model version (*C*_i_-based), *A*_c_ and *A*_j_ are given by:

|  | $A_{c}= \frac{V_{cmax,Ci} \left( C_{i}- \Gamma_{Ci}^{*} \right)}{C_{i}+ K_{c,Ci}\left( 1+ O_{i}/K_{o,Ci} \right)}-R_{l}$ | (S2) |
| --- | --- | --- |

and

|  | $A_{j}= \frac{J\left( C_{i}- \Gamma_{Ci}^{*} \right)}{4C_{i}+8\Gamma_{Ci}^{*}}-R_{l}$ | (S3) |
| --- | --- | --- |

where *V*_cmax,Ci_ is the apparent (*C*_i_-based) maximum carboxylation rate (μmol m^-2^ s^-1^), *C*_i_ and *O*_i_ are the intercellular CO_2_ and O_2_ concentrations (μmol mol^-1^), respectively, $\Gamma_{Ci}^{*}$ is the *C*_i_-based photorespiratory CO_2_ compensation point (μmol mol^-1^), *K*_c,Ci_ and *K*_o,Ci_ are the C_i_-based Michaelis-Menten constants for CO_2_, and O_2_ (μmol mol^-1^), respectively, and *R*_l_ is mitochondrial respiration in the light (μmol m^-2^ s^-1^). See Table S1 for parameter values.

The electron transport rate *J* (μmol m^-2^ s^-1^) is calculated as:

|  | $J= \frac{Q_{a,PSII}+ J_{\max}-\sqrt{{{(Q}_{a,PSII}+J_{\max})}^{2}-4\varepsilon Q_{a,PSII} J_{\max}}}{2\varepsilon}$ | (S4) |
| --- | --- | --- |

where $Q_{a,PSII}$ is absorbed photosynthetic photon flux density by photosystem II (μmol m^-2^ s^-1^), *J*_max_ is the maximum electron transport rate (μmol m^-2^ s^-1^), and ε (0.7) is a curvature parameter.

In the explicit model versions (*C*_c_-based), *A*_c_ and *A*_j_ are calculated with the same formulations, but with all *C*_i_-based values replaced by *C*_c_-based values:

|  | $A_{c}= \frac{V_{cmax,Cc} \left( C_{c}- \Gamma_{Cc}^{*} \right)}{C_{c}+ K_{c,Cc}\left( 1+ O_{c}/K_{o,Cc} \right)}-R_{l}$ | (S5) |
| --- | --- | --- |

and

|  | $A_{j}= \frac{J\left( C_{c}- \Gamma_{Cc}^{*} \right)}{4C_{c}+8\Gamma_{Cc}^{*}}-R_{l}$ | (S6) |
| --- | --- | --- |

where *C*_c_ and *O*_c_ is the concentration of CO_2_ and O_2_, respectively, in the chloroplasts (μmol mol^-1^), $\Gamma_{Cc}^{*}$ is the *C*_c_-based photorespiratory CO_2_ compensation point (μmol mol^-1^), *K*_c,Cc_ and *K*_o,Cc_ are the *C*_c_-based Michaelis-Menten constants for CO_2_, and O_2_ (μmol mol^-1^), respectively (see Table S1 for parameter values). *J* is calculated according to Eq. (S4). *O*_c_ is assumed to equal *O*_i_. C_c_ is given by:

|  | $C_{c}= C_{i}- \frac{A_{n}}{g_{m}}$ | (S7) |
| --- | --- | --- |

In Eq. (S7), mesophyll conductance to CO_2_ transfer (*g*_m_) is calculated a priori according to Eq. (1). Since *A*_n_ occurs in both Eqs. (S1) and (S7), *A*_n_, *C*_i_, and *C*_c_ are solved numerically in iteration loops.
