# Supplementary material for: Effects of mesophyll conductance on vegetation responses to elevated CO2 concentrations in a land surface model
Source: Glob Chang Biol. 2019 Mar 23;25(5):1820–38. doi: 10.1111/gcb.14604 (PMC6487956; doi:10.1111/gcb.14604)
Supplement: Supplementary file 2 [file GCB-25-1820-s002.docx]

## Appendix S2 Literature survey on mesophyll conductance (*g*_m_) and data processing.

The literature survey was conducted using Google Scholar. Search terms were “mesophyll conductance” and “internal conductance”. The search included peer-reviewed literature published online until 31.12.2017 (with the exception of Bahar *et al.* (2018))

Only studies were used in which *g*_m_ is defined as *g*_m_ = *A*_n_/(*C*_i_-*C*_c_). Alternative definitions such as *g*_m_ = *A*_n_/*C*_i_ or *g*_m_ as the initial slope of the *A*_n_/*C*_i_ curve were excluded. Reported values represent unstressed, fully expanded, non-senescent, young leaves exposed to full sun (note that not always all of these criteria (except the first one) were stated explicitly and had to be assumed for some studies). If data were presented in figures, individual data points were retrieved from the graphs using Plot Digitizer (version 2.6.8, Joseph A. Huwaldt). In a few cases, *g*_m_ was calculated from *A*_n_, *C*_i_ and *C*_c_ using *g*_m_ = *A*_n_/(*C*_i_-*C*_c_).

If units were reported in pressure units (mol m^-2^ s^-1^ bar^-1^ or μmol m^-2^ s^-1^ Pa^-1^), they were converted to mol m^-2^ s^-1^ using atmospheric pressure (*P*) at the measurement location. If *P* was not reported, it was estimated from the hypsometric equation using the altitude of the measurement location. If altitude was not reported or below 200 m, *P* was assumed to equal approx. 100 kPa (=1 bar) and units were not converted.

*g*_m_ was standardized to 25 °C using Eq. 2 with parameter values from Bernacchi *et al.* (2002). In cases where leaf temperature was not reported, cuvette temperature was taken as a proxy. Measurements for which temperature was not reported were discarded. In case of model versions *ExpL* and *ExpCL*, which assume a light dependency of *g*_m_, *g*_m_ was standardized to high light (1500 μmol m^-2^ s^-1^) using Eq. 6.

Records obtained with all measurement methods were included in the analysis. If g_m_ was measured with both the curve fitting technique and an additional method (e.g. gas exchange and chlorophyll fluorescence or gas exchange and carbon isotope discrimination), only the additional method was taken, as they are considered more reliable than the curve fitting method (Pons *et al.*, 2009).

A basic outlier removal procedure was implemented at PFT-level. Records were flagged as outliers and removed if the distance of *g*_m_ values exceeded 1.5 times the interquartile range of the log-transformed *g*_m_ data within each PFT. The log-transformation was necessary to obtain normally distributed data, which enabled the detection of both high and low outliers. This procedure led to the exclusion of 23 records (3.6%).

In total 325 studies were analyzed which contained 821 measurements from 407 species. Among them, 319 species from 295 studies and 609 individual measurements fulfilled all criteria described above and could be assigned to a PFT considered by JSBACH. These measurements (see Fig. 1; Appendix S3) were then used to parameterize the model.

**References**

Bahar NH, Hayes L, Scafaro AP, Atkin OK, Evans JR (2018) Mesophyll conductance does not contribute to greater photosynthetic rate per unit nitrogen in temperate compared with tropical evergreen wet-forest tree leaves. *New Phytologist,* **218**, 492-505.

Bernacchi CJ, Portis AR, Nakano H, von Caemmerer S, Long SP (2002) Temperature response of mesophyll conductance. Implications for the determination of Rubisco enzyme kinetics and for limitations to photosynthesis in vivo. *Plant Physiology,* **130**, 1992-1998.

Pons TL, Flexas J, Von Caemmerer S, Evans JR, Genty B, Ribas-Carbo M, Brugnoli E (2009) Estimating mesophyll conductance to CO_2_: methodology, potential errors, and recommendations. *Journal of Experimental Botany,* **60**, 2217-2234.
