# Supplementary material for: Effects of mesophyll conductance on vegetation responses to elevated CO2 concentrations in a land surface model
Source: Glob Chang Biol. 2019 Mar 23;25(5):1820–38. doi: 10.1111/gcb.14604 (PMC6487956; doi:10.1111/gcb.14604)
Supplement: Supplementary file 4 [file GCB-25-1820-s004.docx]

**Supplementary Information for Knauer et al.,**

**Global Change Biology**

**Article title:** “Effects of mesophyll conductance on vegetation responses to elevated CO_2_ concentrations in a land surface model”

**Authors:** Jürgen Knauer, Sönke Zaehle, Martin G. De Kauwe, Nur H.A. Bahar, John R. Evans,

Belinda E. Medlyn, Markus Reichstein, Christiane Werner

**Accompanying files:**

Appendix S1: Calculation of net assimilation in the JSBACH model.

Appendix S2: Literature survey on mesophyll conductance (*g*_m_) and data processing.

Appendix S3: Mesophyll conductance dataset.

**Table S1**: *C*_i_- and *C*_c_-based photosynthetic parameters used in this study.

| Vegetation type | Parameter | ***C*_i_-based** | | | |  | ***C*_c_-based** | | | |
| --- | --- | --- | --- | --- | --- | --- | --- | --- | --- | --- |
|  |  | Value at 25°C | *H*_a_  (kJ mol^-1^) | *H*_d_  (kJ mol^-1^) | $\Delta S$  (J mol^-1^ K^-1^) |  | Value at 25°C | *H*_a_  (kJ mol^-1^) | *H*_d_  (kJ mol^-1^) | $\Delta S$  (J mol^-1^ K^-1^) |
| C3 vegetation | *g*_m,max_ (mol m^-2^ s^-1^) | - | **-** | - | - |  | PFT-specific | 49.60 | 437.4 | 1400 |
|  | *V*_cmax_ (μmol m^-2^ s^-1^) | PFT-specific | 65.33 | 200.0 | 635 |  | PFT-specific | 65.33 | 200.0 | 635 |
|  | *J*_max_ (μmol m^-2^ s^-1^) | PFT-specific | 43.90 | 200.0 | 640 |  | PFT-specific | 43.90 | 200.0 | 640 |
|  | *R*_l_ (μmol m^-2^ s^-1^) | 0.011V_cmax25,Ci_ | 46.39 | 200.0 | 640 |  | 0.011 *V*_cmax25,Ci_ | 46.39 | 200.0 | 640 |
|  | *K*_c_ (μmol mol^-1^) | 404.9 | 79.43 | - | - |  | 272.38 | 80.99 | - | - |
|  | *K*_o_ (mmol mol^-1^) | 278.4 | 36.38 | - | - |  | 165.82 | 23.72 | - | - |
|  | *Γ**(μmol mol^-1^) | 42.75 | 37.83 | - | - |  | 37.43 | 24.46 | - | - |
| C4 vegetation | *g*_m,max_ (mol m^-2^ s^-1^) | - | **-** | - | - |  | PFT-specific | 49.60 | 437.4 | 1400 |
|  | *V*_cmax_ (μmol m^-2^ s^-1^) | PFT-specific | 67.29 | 144.6 | 472 |  | PFT-specific | 67.29 | 144.6 | 472 |
|  | *J*_max_ (μmol m^-2^ s^-1^) | PFT-specific | 77.90 | 191.9 | 627 |  | PFT-specific | 77.90 | 191.9 | 627 |
|  | *V*_pmax_ (μmol m^-2^ s^-1^) | PFT-specific | 70.37 | 117.9 | 376 |  | PFT-specific | 70.37 | 117.9 | 376 |
|  | *V_pr_* (μmol m^-2^ s^-1^)^a^ | 0.67 *V*_pmax25,Ci_ | - | - | - |  | 0.67 *V*_pmax25,Ci_ | - | - | - |
|  | *R*_l_ (μmol m^-2^ s^-1^) | 0.01 *V*_cmax25,Ci_ | 46.39 | 200.0 | 640 |  | 0.01 *V*_cmax25,Ci_ | 46.39 | 200.0 | 640 |
|  | *K*_p_ (μmol mol^-1^)^b^ | 80.0 | 68.10 | - | - |  | 80.0 | 68.10 | - | - |
|  | *g*_bs_ (mol m^-2^ s^-1^)^c^ | 0.003 | - | - | - |  | 0.003 | - | - | - |

^a^ *V*_pr_ = PEP regeneration rate

^b^ *K*_p_ = Michaelis-Menten constant of PEP-carboxylase for CO_2_

^c^ *g*_bs_ = bundle-sheath conductance to CO_2_

**Table S2:** As Table 3, but for the *ExpL* and *ExpCL* model versions.

| PFT | *g*_m,max25_^a^ ± SEM^b^  (mol m^-2^ s^-1^) | *V*_cmax25,Cc_  (μmol m^-2^ s^-1^) | *J*_max25,Cc_  (μmol m^-2^ s^-1^) | *J*_max25,Cc_ / *V*_cmax25,Cc_ | *g*_m,max25_^c^  (mol m^-2^ s^-1^) | *V*_cmax25,Cc_  (μmol m^-2^ s^-1^) | *J*_max25,Cc_  (μmol m^-2^ s^-1^) | *J*_max25,Cc_ / *V*_cmax25,Cc_ |
| --- | --- | --- | --- | --- | --- | --- | --- | --- |
|  | *ExpL* | *ExpL* | *ExpL* | *ExpL* | *ExpCL* | *ExpCL* | *ExpCL* | *ExpCL* |
| DNF | 0.060 ± 0.010 | 55.3 | 64.7 | 1.17 | 0.056 | 64.1 | 85.1 | 1.33 |
| TDF | 0.062 ± 0.022 | 52.7 | 64.3 | 1.22 | 0.061 | 49.6 | 70.4 | 1.42 |
| ENF | 0.088 ± 0.022 | 98.8 | 103.8 | 1.05 | 0.083 | 116.6 | 152.1 | 1.30 |
| DSH | 0.113 ± 0.026 | 67.1 | 96.1 | 1.43 | 0.114 | 70.2 | 106.6 | 1.52 |
| EBF | 0.109 ± 0.010 | 105.4 | 120.2 | 1.14 | 0.107 | 113.6 | 152.6 | 1.34 |
| TRF | 0.156 ± 0.027 | 41.9 | 74.0 | 1.77 | 0.151 | 43.7 | 76.9 | 1.76 |
| DBF | 0.187 ± 0.017 | 58.6 | 99.3 | 1.69 | 0.184 | 60.0 | 103.6 | 1.73 |
| C3G | 0.201 ± 0.015 | 53.7 | 95.1 | 1.77 | 0.202 | 55.5 | 98.5 | 1.78 |
| RSH | 0.228 ± 0.112 | 52.0 | 94.3 | 1.82 | 0.235 | 53.2 | 97.0 | 1.82 |
| C3C | 0.312 ± 0.018 | 86.6 | 152.3 | 1.76 | 0.323 | 88.9 | 157.8 | 1.77 |
|  |  | *V*_pmax25,Cc_  (μmol m^-2^ s^-1^) |  |  |  | *V*_pmax25,Cc_  (μmol m^-2^ s^-1^) |  |  |
| C4G | 0.453 ± 0.154 | 118.3 |  |  | 0.382 | 189.0 |  |  |
| C4C | 0.743 ± 0.477 | 142.7 |  |  | 0.623 | 185.6 |  |  |

^a^ standardized to a *C*_i_ of 260 μmol mol^-1^ (Eq. (5))

^b^ SEM = standard error of the median

^c^ standardized to a *Q*_a_ of 1500 μmol m^-2^ s^-1^ (Eq. (6))

**Table S3:** Goodness of fit metrics for simulated *A*_n_ of the explicit (*Exp*) model version compared to the implicit (*Imp*) model over a *C*_i_ range of 0-1500 μmol mol^-1^. Other parameters were: *V*_cmax25,Ci_ = 40 μmol m^-2^ s^-1^, *J*_max25,Ci_ = 76 μmol m^-2^ s^-1^.

| *g*_m,max25_  (mol m^-2^ s^-1^) | RMSE ^a^  (μmol m^-2^ s^-1^) | MAE ^b^  (μmol m^-2^ s^-1^) |
| --- | --- | --- |
| 0.300 | 0.056 | 0.025 |
| 0.200 | 0.073 | 0.051 |
| 0.150 | 0.123 | 0.097 |
| 0.100 | 0.249 | 0.202 |
| 0.075 | 0.416 | 0.334 |

^a^ RMSE = Root mean square error

^b^ MAE = Mean absolute error

**Table S4:** Sensitivity of the parameter adjustment approach to different Rubisco kinetic parameter sets as derived by **Bernacchi *et al.* (2001) and Bernacchi *et al.* (2002)** as used in this study (K_c,Ci_ = 404.9 μmol mol^-1^; K_o,Ci_ = 278.4 mmol mol^-1^; $\Gamma_{Ci}^{*}=$ 42.75 μmol mol^-1^, K_c,Cc_ = 272.38 μmol mol^-1^; K_o,Cc_ = 165.82 mmol mol^-1^; $\Gamma_{Cc}^{*}=$ 37.43 μmol mol^-1^)*,* **von Caemmerer *et al.* (1994)** (K_c,Ci_ = 404 μmol mol^-1^; K_o,Ci_ = 248 mmol mol^-1^; $\Gamma_{Ci}^{*}=$ 36.9 μmol mol^-1^; K_c,Cc_ = 259 μmol mol^-1^; K_o,Cc_ = 179 mmol mol^-1^; $\Gamma_{Cc}^{*}=$ 38.6 μmol mol^-1^ (at a pressure of 953 hPa)), and **Walker *et al*. (2013)** (*C*_i_-based parameters assumed to be as in Bernacchi et al. 2001, K_c,Cc_ = 315 μmol mol^-1^; K_o,Cc_ = 215 mmol mol^-1^; $\Gamma_{Cc}^{*}=$ 40 μmol mol^-1^ (at measurement pressure)). The parameter adjustment was made for a hypothetical leaf with the following photosynthetic characteristics: *g*_m,max25_ = 0.15 mol m^-2^ s^-1^; V_cmax25,Ci_ = 50 μmol m^-2^ s^-1^; J_max25,Ci_ = 95 μmol m^-2^ s^-1^; V_cmax25,Ci_/J_max25,Ci_ = 1.9; R_l_ = 0.5 μmol m^-2^ s^-1^.

| Study | V_cmax25,Cc_  (μmol m^-2^ s^-1^) | J_max25,Cc_  (μmol m^-2^ s^-1^) | V_cmax25,Cc_/J_max25,Cc_ |
| --- | --- | --- | --- |
| Bernacchi *et al.* (2001, 2002) | 58.53 | 95.53 | 1.63 |
| Walker *et al.* (2013) | 60.20 | 96.46 | 1.60 |
| von Caemmerer *et al.* (1994) | 54.11 | 97.58 | 1.80 |


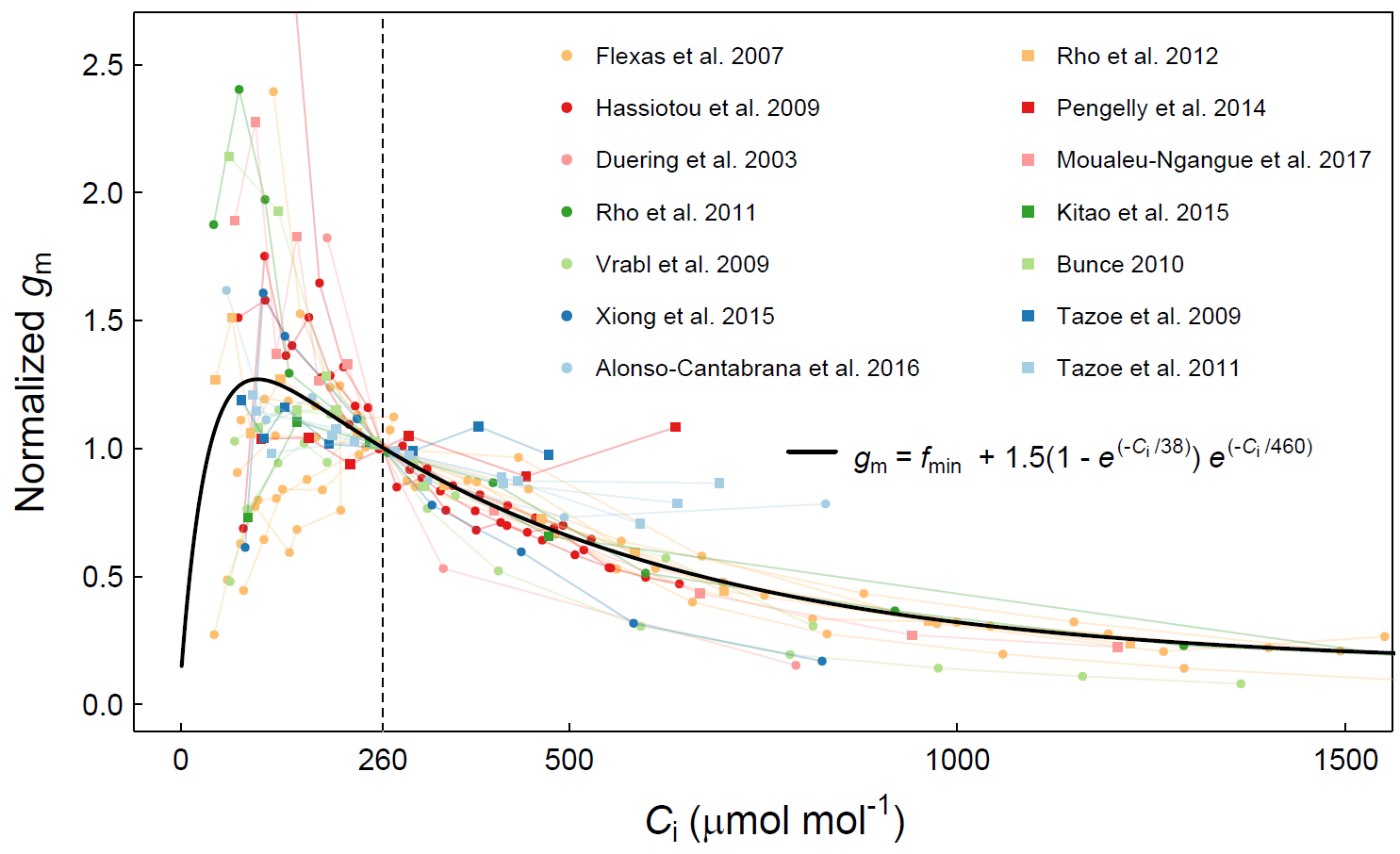


**Figure S1** Compilation of published *C*_i_ (intercellular CO_2_ concentration) responses of *g*_m_. *g*_m_ was normalized to a value of 1 at *C*_i_ of 260 μmol mol^-1^. The bold black line illustrates the function as implemented in the model (Eq. 5) with f_min_ = 0.15.


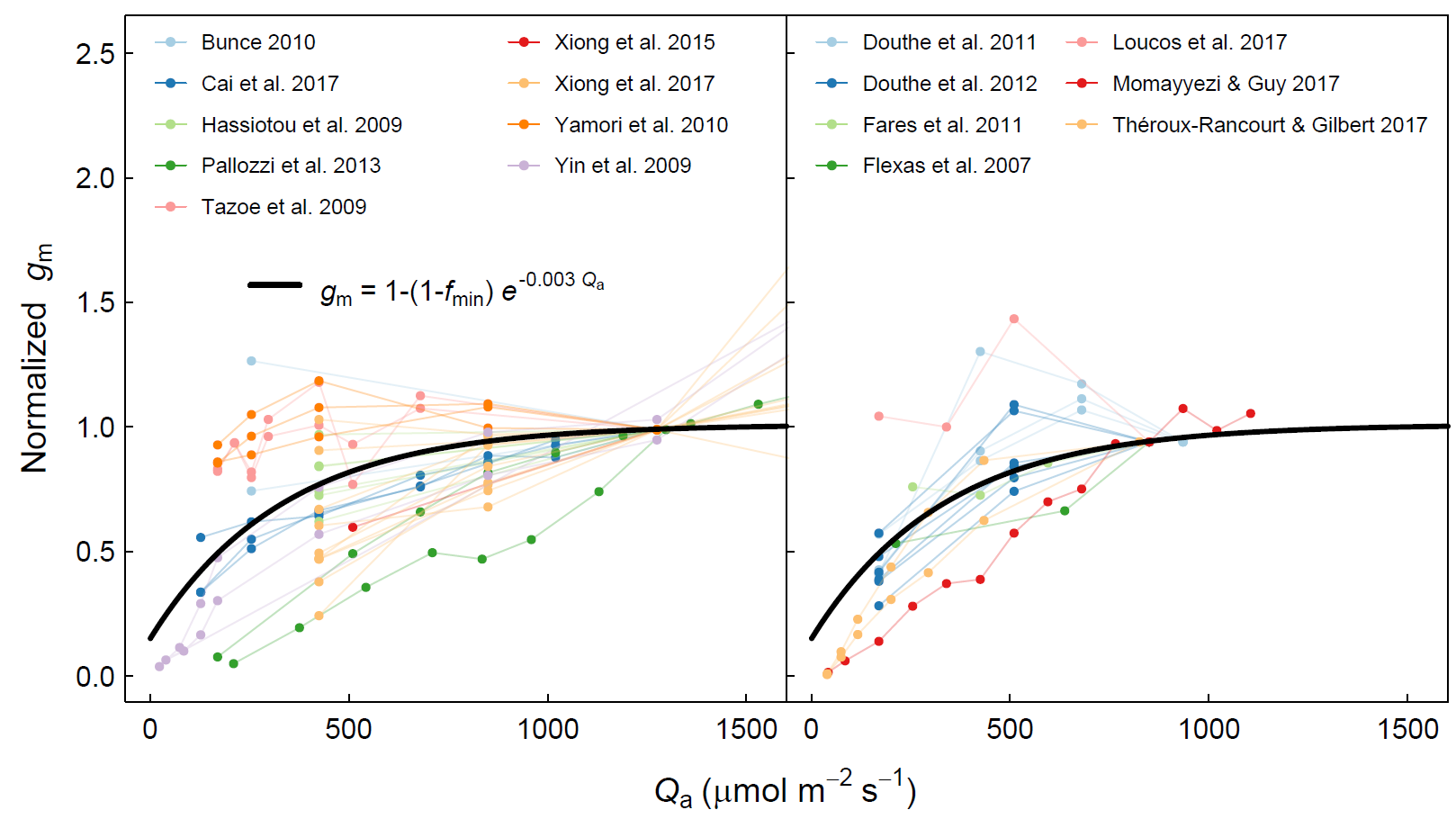


**Figure S2** Compilation of published light responses of *g*_m_. *g*_m_ was normalized to *Q*_a_ (absorbed photosynthetic photon flux density (Q)) of 1250 μmol m^-2^ s^-1^ (left) and 850 μmol m^-2^ s^-1^ (right). *Q*_a_ was assumed to equal 0.85·Q. The bold black line illustrates the function (Eq. 6) as implemented in the *g*_m_ model of this study (Eq. 1).


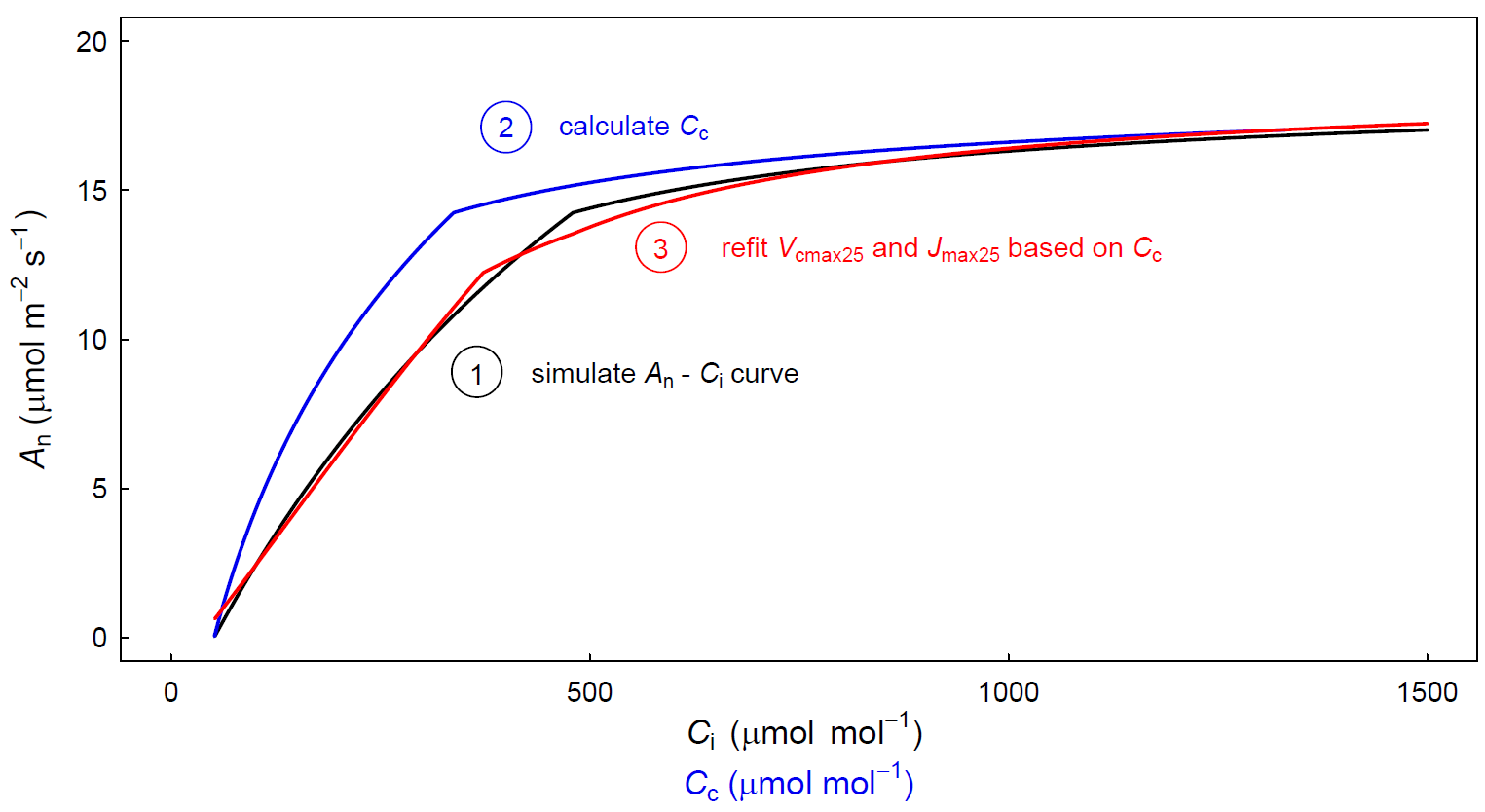


**Figure S3** Illustration of the parameter adjustment procedure implemented in this study for the C3 photosynthesis model of Farquhar *et al.* (1980). Parameters of the *C*_i_-based model (step 1) are: *V*_cmax25,Ci_ = 40 μmol m^-2^ s^-1^; *J*_max25,Ci_ = 76 μmol m^-2^ s^-1^; *R*_l_ = 0.44 μmol m^-2^ s^-1^; Rubisco kinetic parameters are listed in Table S1. *g*_m,max25_ = 0.1 mol m^-2^ s^-1^ and is assumed to be independent of *C*_i_ (step 2). The resulting *C*_c_-based parameters are (step 3): *V*_cmax25,Cc_ = 50.8 μmol m^-2^ s^-1^; *J*_max25,Cc_ = 76.9 μmol m^-2^ s^-1^.


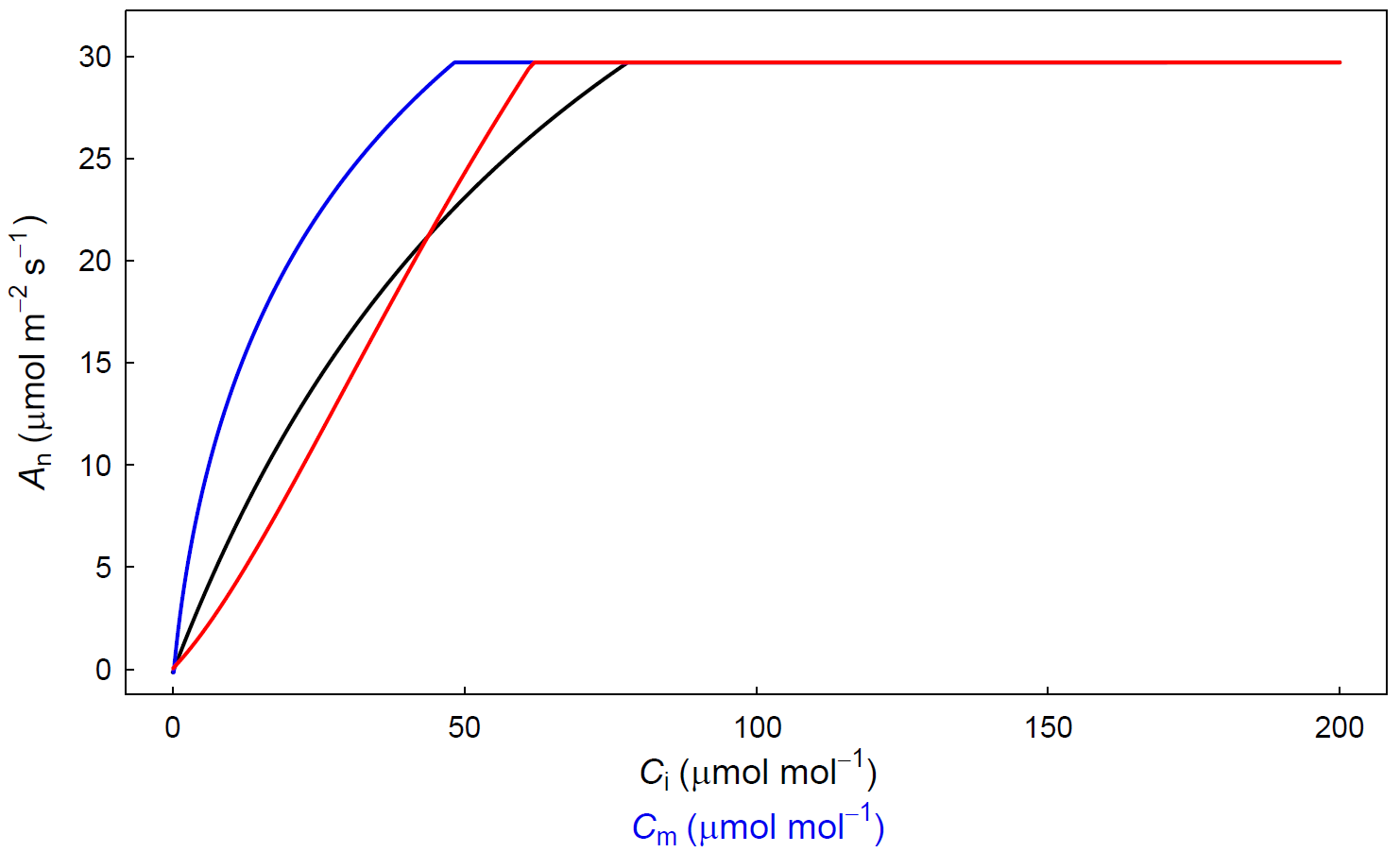


**Figure S4** Same as Figure S3 but for the C4 photosynthesis model of von Caemmerer and Furbank (1999). Parameters of the *C*_i_-based model (black line) are: *V*_cmax25,Ci_ = 30 μmol m^-2^ s^-1^; *J*_max25,Ci_ = 200 μmol m^-2^ s^-1^; *V*_pmax25,Ci_ = 60 μmol m^-2^ s^-1^; *V*_pr_ = 40 μmol m^-2^ s^-1^, *R*_l_ = 0.3 μmol m^-2^ s^-1^. *g*_m,max25_ = 1 mol m^-2^ s^-1^ and is assumed to be independent of *C*_i_. The resulting *C*_c_-based parameters (red line) are: *V*_pmax25,Cc_ = 96.7 μmol m^-2^ s^-1^ and all other parameters are unchanged.


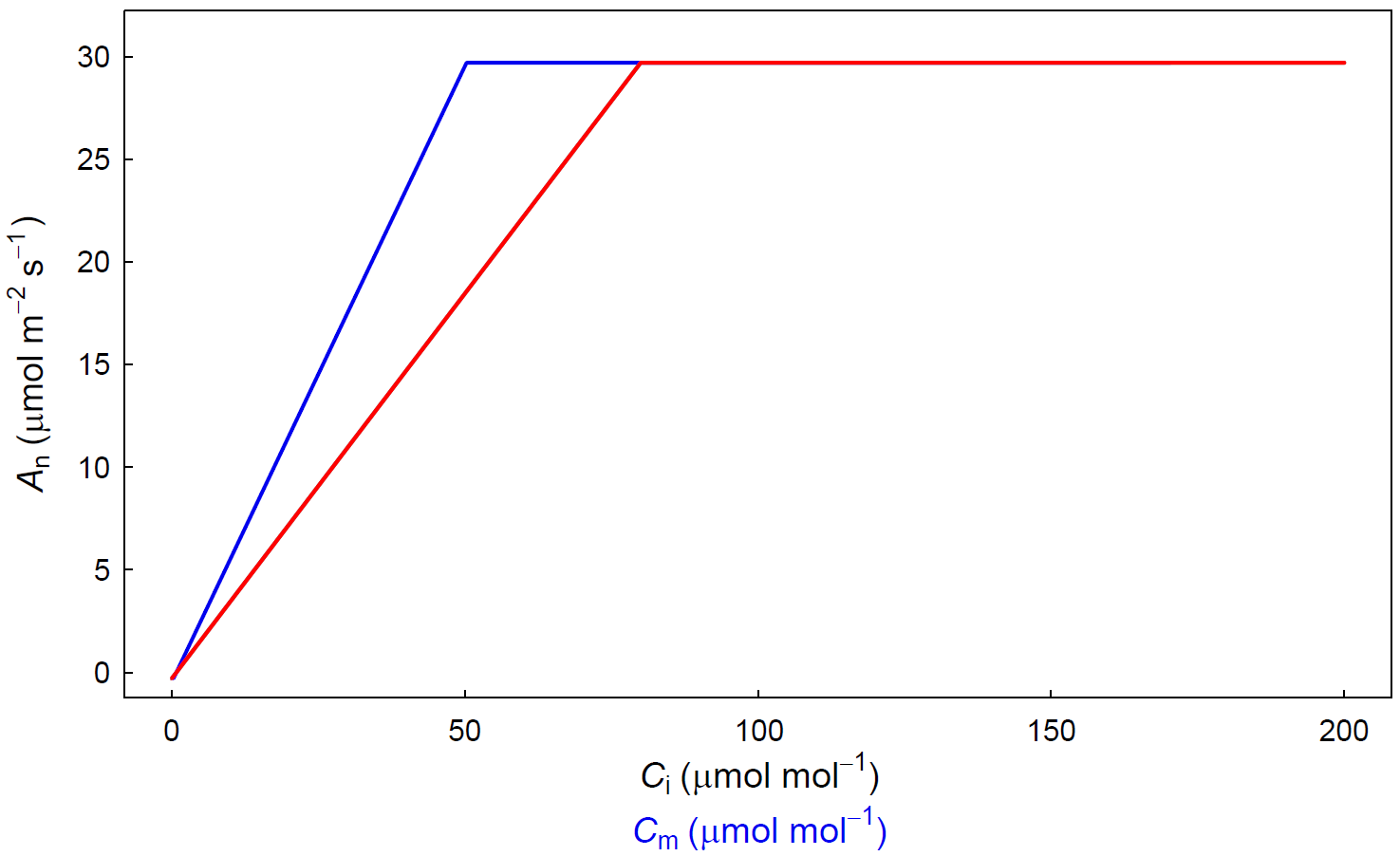


**Figure S5** Same as Figure S3 but for the C4 photosynthesis model of Collatz *et al.* (1992) as implemented in Bonan *et al.* (2011). Parameter values were *V*_cmax25,Ci_ = 30 μmol m^-2^ s^-1^; *k*_Ci_ (initial slope of the CO_2_ response curve) = 0.375; *g*_m_ = 1 mol m^-2^ s^-1^;. The resulting *k*_Cc_ = 0.258. For this model, the *C*_i_-based and *C*_c_-based model simulations are identical.


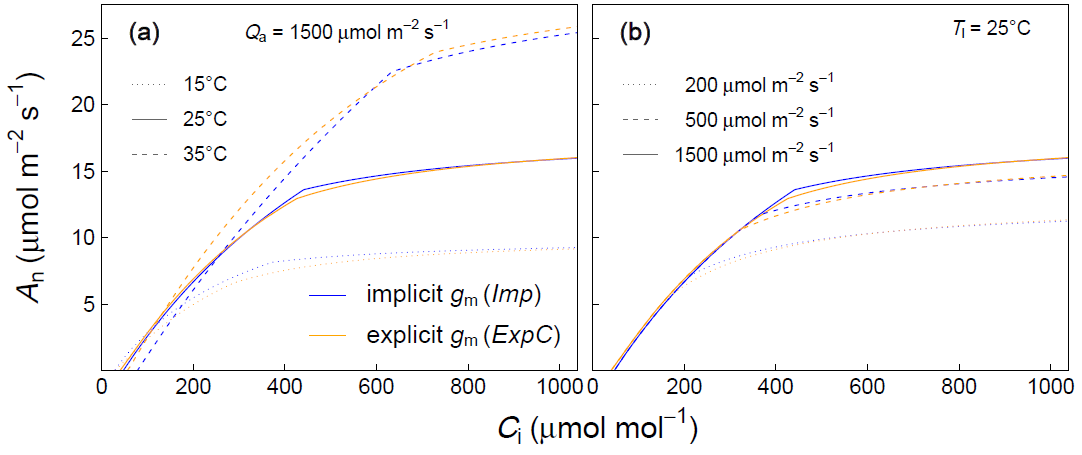


**Figure S6** Same as Figure 2a,b, but for the *ExpC* model version (*g*_m_ assumed to respond to intercellular CO_2_ concentration (*C*_i_) according to Eq. 5).


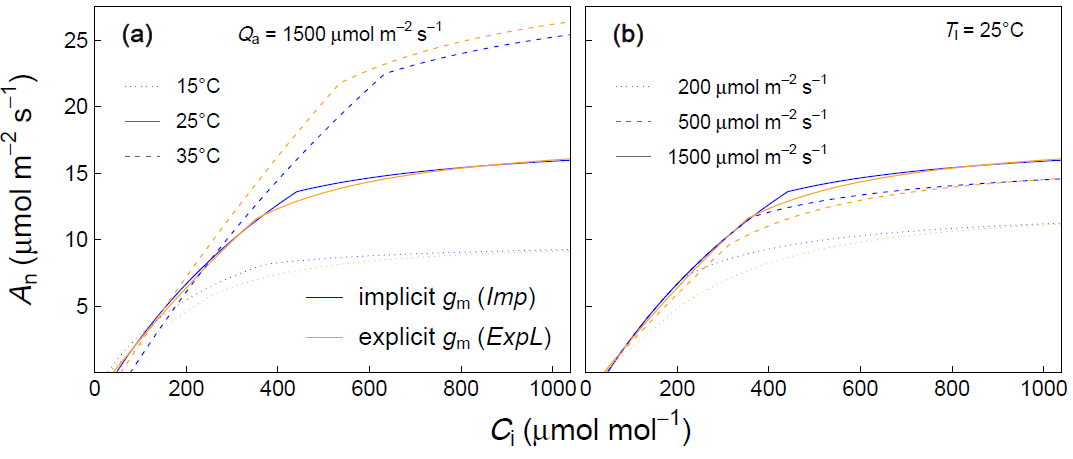


**Figure S7** Same as Figure 2a,b, but for the *ExpL* model version (*g*_m_ assumed to respond to absorbed photosynthetic photon flux density (*Q*_a_) according to Eq. 6).


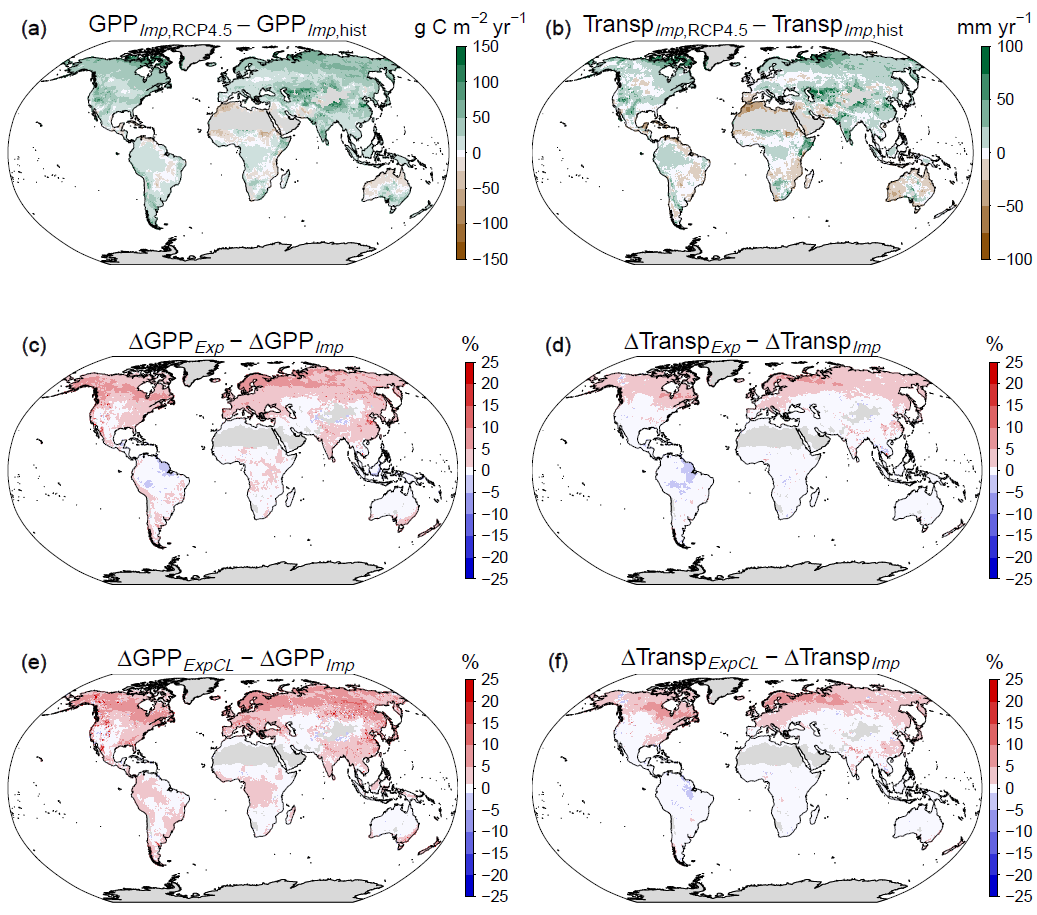


**Figure S8** Same as Figure 6, but for the RCP4.5 scenario.


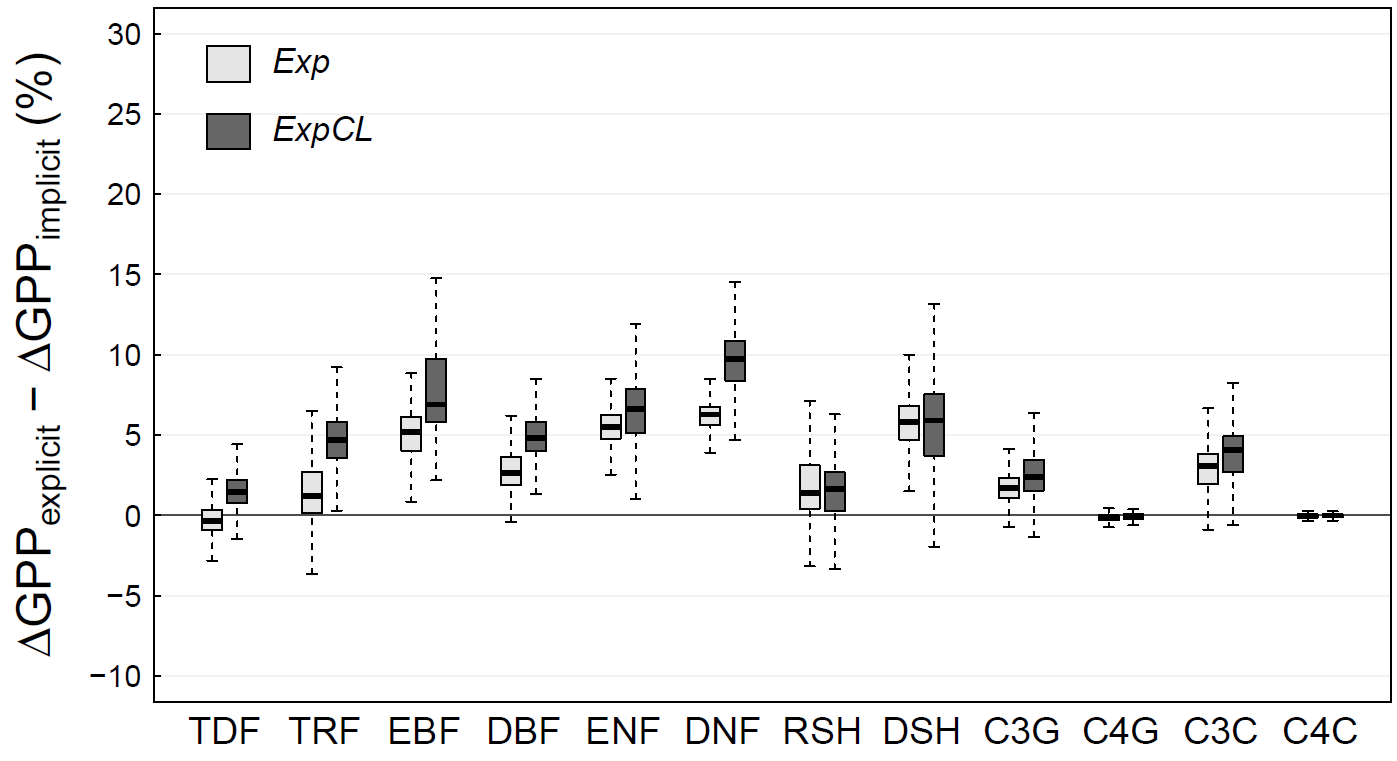


## Figure S9 Same as Figure 7, but for the RCP4.5 scenario.


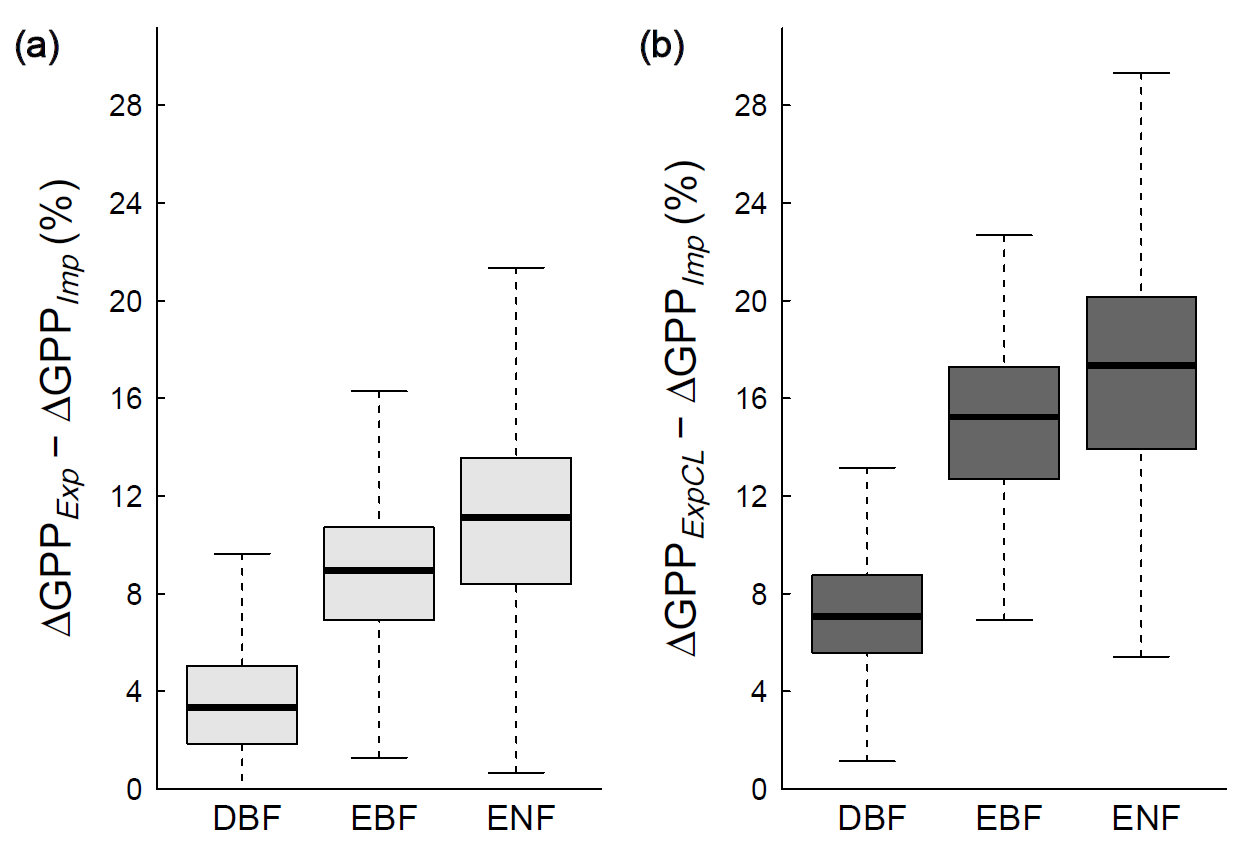


**Figure S10** Same as Figure 7, but for the three co-occurring (i.e. >5% fractional cover in the same grid cell) plant functional types deciduous broadleaf trees (DBF), evergreen broadleaf trees (EBF), and evergreen needle-leaf trees (ENF) only.


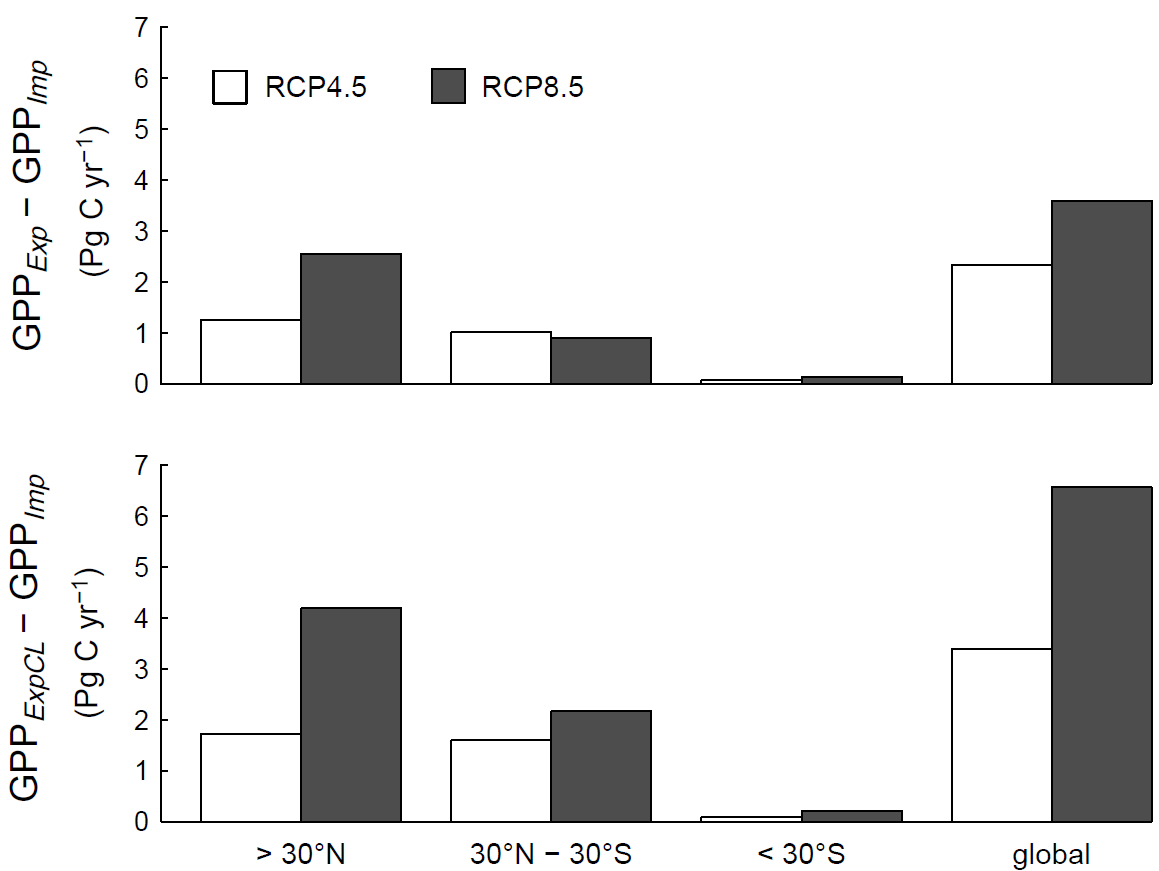


**Figure S11** Differences in mean annual gross primary productivity (GPP) in the RCP4.5 and RCP8.5 scenarios (2070-2099) for different latitudinal bands and globally.


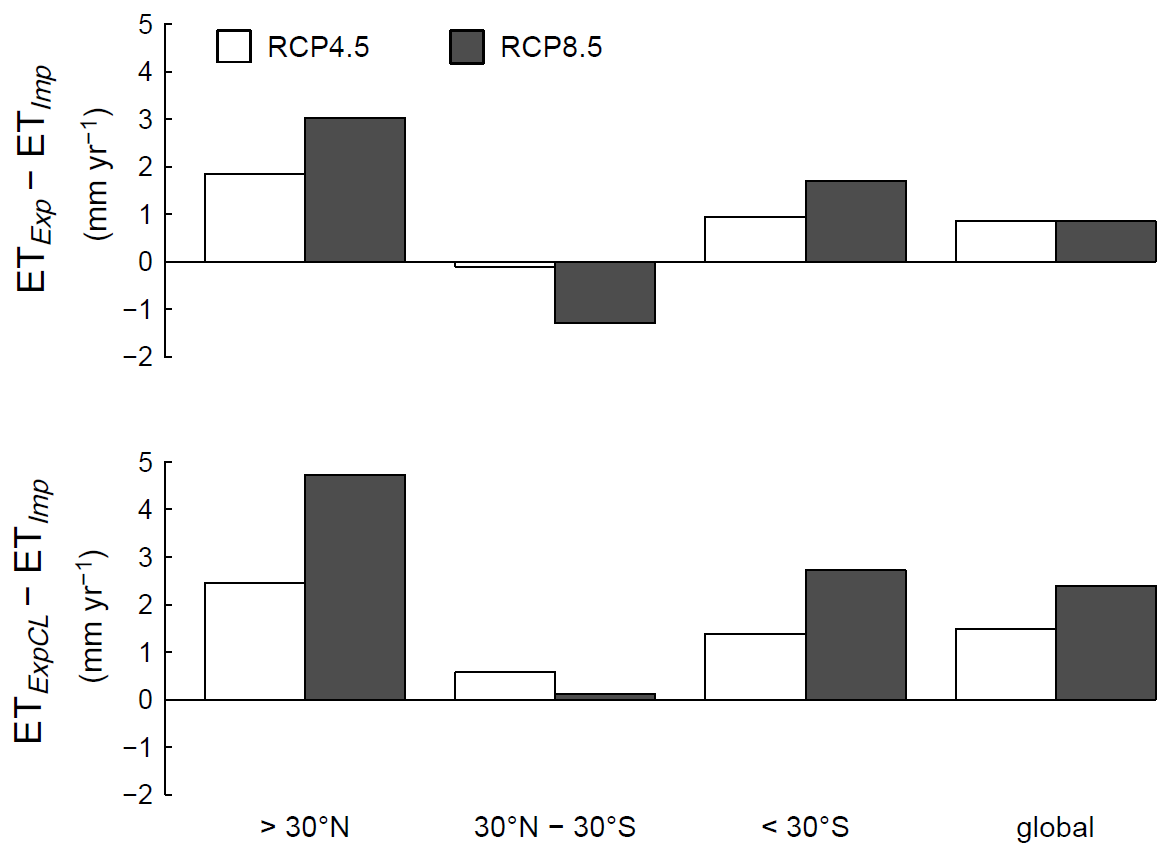


**Figure S12** Differences in mean annual evapotranspiration (ET) in the RCP4.5 and RCP8.5 scenarios (2070-2099) for different latitudinal bands and globally.


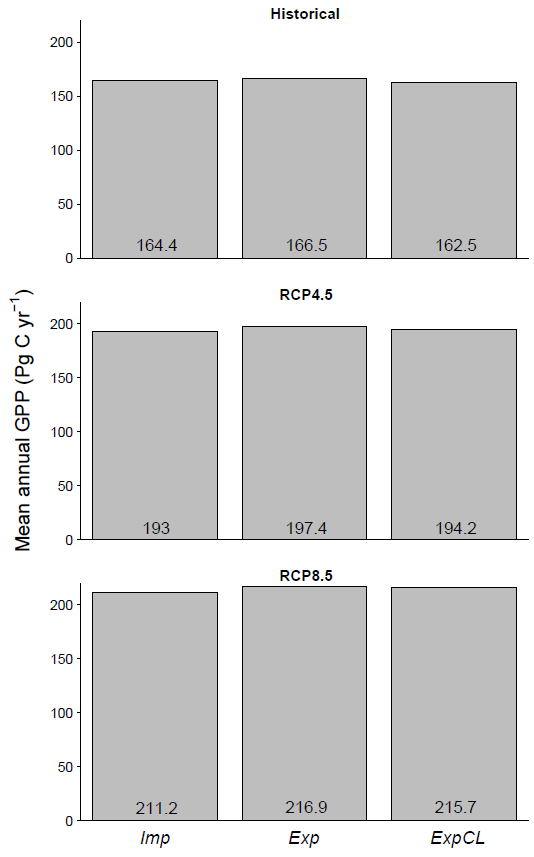


**Figure S13** Global mean annual gross primary productivity (GPP) for model versions and scenarios used in this study. ‘Historical’ refers to the time period 1975-2004, ‘RCP4.5’ and ‘RCP8.5’ refer to the time period 2070-2099.

**References**

Alonso-Cantabrana H, von Caemmerer S (2016) Carbon isotope discrimination as a diagnostic tool for C4 photosynthesis in C3-C4 intermediate species. *Journal of Experimental Botany,* **67**, 3109-3121.

Bahar NH, Hayes L, Scafaro AP, Atkin OK, Evans JR (2018) Mesophyll conductance does not contribute to greater photosynthetic rate per unit nitrogen in temperate compared with tropical evergreen wet-forest tree leaves. *New Phytologist,* **218**, 492-505.

Bernacchi CJ, Portis AR, Nakano H, von Caemmerer S, Long SP (2002) Temperature response of mesophyll conductance. Implications for the determination of Rubisco enzyme kinetics and for limitations to photosynthesis in vivo. *Plant Physiology,* **130**, 1992-1998.

Bernacchi CJ, Singsaas EL, Pimentel C, Portis AR, Long SP (2001) Improved temperature response functions for models of Rubisco-limited photosynthesis. *Plant, Cell & Environment*, **24**, 253-259.

Bonan GB, Lawrence PJ, Oleson KW *et al.* (2011) Improving canopy processes in the Community Land Model version 4 (CLM4) using global flux fields empirically inferred from FLUXNET data. *Journal of Geophysical Research: Biogeosciences,* **116**.

Bunce J (2010) Variable responses of mesophyll conductance to substomatal carbon dioxide concentration in common bean and soybean. *Photosynthetica,* **48**, 507-512.

Collatz GJ, Ribas-Carbo M, Berry J (1992) Coupled photosynthesis-stomatal conductance model for leaves of C4 plants. *Functional Plant Biology,* **19**, 519-538.

Douthe C, Dreyer E, Brendel O, Warren CR (2012) Is mesophyll conductance to CO_2_ in leaves of three Eucalyptus species sensitive to short-term changes of irradiance under ambient as well as low O_2_? *Functional Plant Biology,* **39**, 435-448.

Douthe C, Dreyer E, Epron D, Warren CR (2011) Mesophyll conductance to CO_2_, assessed from online TDL-AS records of ^13^CO_2_ discrimination, displays small but significant short-term responses to CO_2_ and irradiance in *Eucalyptus* seedlings. *Journal of Experimental Botany,* **62**, 5335-5346.

Düring H (2003) Stomatal and mesophyll conductances control CO_2_ transfer to chloroplasts in leaves of grapevine (*Vitis vinifera* L.). *VITIS-Journal of Grapevine Research,* **42**, 65.

Fares S, Mahmood T, Liu S, Loreto F, Centritto M (2011) Influence of growth temperature and measuring temperature on isoprene emission, diffusive limitations of photosynthesis and respiration in hybrid poplars. *Atmospheric Environment,* **45**, 155-161.

Farquhar G, von Caemmerer S, Berry J (1980) A biochemical model of photosynthetic CO_2_ assimilation in leaves of C3 species. *Planta,* **149**, 78-90.

Flexas J, Diaz-Espejo A, Galmés J, Kaldenhoff R, Medrano H, Ribas-Carbo M (2007) Rapid variations of mesophyll conductance in response to changes in CO_2_ concentration around leaves. *Plant, Cell & Environment,* **30**, 1284-1298.

Kitao M, Yazaki K, Kitaoka S *et al.* (2015) Mesophyll conductance in leaves of Japanese white birch (*Betula platyphylla* var. *japonica*) seedlings grown under elevated CO_2_ concentration and low N availability. *Physiologia plantarum,* **155**, 435-445.

Loucos KE, Simonin KA, Barbour MM (2017) Leaf hydraulic conductance and mesophyll conductance are not closely related within a single species. *Plant, Cell & Environment,* **40**, 203-215.

Momayyezi M, Guy RD (2017) Blue light differentially represses mesophyll conductance in high vs low latitude genotypes of *Populus trichocarpa* Torr. & Gray. *Journal of Plant Physiology,* **213**, 122-128.

Moualeu-Ngangue DP, Chen T-W, Stützel H (2017) A new method to estimate photosynthetic parameters through net assimilation rate- intercellular space CO_2_ concentration (A-Ci) curve and chlorophyll fluorescence measurements. *New Phytologist,* **213**, 1543-1554.

Pallozzi E, Tsonev T, Marino G, Copolovici L, Niinemets Ü, Loreto F, Centritto M (2013) Isoprenoid emissions, photosynthesis and mesophyll diffusion conductance in response to blue light. *Environmental and Experimental Botany,* **95**, 50-58.

Pengelly J, Förster B, von Caemmerer S, Badger M, Price GD, Whitney S (2014) Transplastomic integration of a cyanobacterial bicarbonate transporter into tobacco chloroplasts. *Journal of Experimental Botany,* **65**, 3071-3080.

Pons TL, Flexas J, Von Caemmerer S, Evans JR, Genty B, Ribas-Carbo M, Brugnoli E (2009) Estimating mesophyll conductance to CO_2_: methodology, potential errors, and recommendations. *Journal of Experimental Botany,* **60**, 2217-2234.

Rho H, Yu DJ, Kim SJ, Chun C, Lee HJ (2011) Estimation of carboxylation efficiency from net CO_2_ assimilation rate as a function of chloroplastic CO_2_ concentration in strawberry (*Fragaria ananassa* cv. Maehyang) leaves. *Horticulture, Environment, and Biotechnology,* **52**, 547-552.

Rho H, Yu DJ, Kim SJ, Lee HJ (2012) Limitation factors for photosynthesis in ‘Bluecrop’ highbush blueberry (*Vaccinium corymbosum*) leaves in response to moderate water stress. *Journal of Plant Biology,* **55**, 450-457.

Tazoe Y, Von Caemmerer S, Estavillo GM, Evans JR (2011) Using tunable diode laser spectroscopy to measure carbon isotope discrimination and mesophyll conductance to CO_2_ diffusion dynamically at different CO_2_ concentrations. *Plant, Cell & Environment,* **34**, 580-591.

von Caemmerer S, Evans JR, Hudson GS, Andrews TJ (1994) The kinetics of ribulose-1, 5-bisphosphate carboxylase/oxygenase in vivo inferred from measurements of photosynthesis in leaves of transgenic tobacco. *Planta,* **195**, 88-97.

von Caemmerer S, Furbank RT (1999) Modeling C4 photosynthesis. In: *C4 Plant Biology,* (eds Sage RF, Monson RK), pp. 173-211*,* Academic Press Toronto, ON, Canada,

Vrábl D, Vasková M, Hronková M, Flexas J, Santrucek J (2009) Mesophyll conductance to CO_2_ transport estimated by two independent methods: effect of variable CO_2_ concentration and abscisic acid. *Journal of Experimental Botany,* **60**, 2315-2323.

Walker B, Ariza LS, Kaines S, Badger MR, Cousins AB (2013) Temperature response of *in vivo* Rubisco kinetics and mesophyll conductance in *Arabidopsis thaliana*: comparisons to *Nicotiana tabacum*. *Plant, Cell & Environment*, **36**, 2108-2119.

Xiong D, Liu X, Liu L, Douthe C, Li Y, Peng S, Huang J (2015) Rapid responses of mesophyll conductance to changes of CO_2_ concentration, temperature and irradiance are affected by N supplements in rice. *Plant, Cell & Environment,* **38**, 2541-2550.

Yamori W, Evans JR, Von Caemmerer S (2010) Effects of growth and measurement light intensities on temperature dependence of CO_2_ assimilation rate in tobacco leaves. *Plant, Cell & Environment,* **33**, 332-343.

Yin X, Struik PC, Romero P, Harbinson J, Evers JB, Van Der Putten PE, Vos J (2009) Using combined measurements of gas exchange and chlorophyll fluorescence to estimate parameters of a biochemical C3 photosynthesis model: a critical appraisal and a new integrated approach applied to leaves in a wheat (*Triticum aestivum*) canopy. *Plant, Cell & Environment,* **32**, 448-464.
